# Influence of Wiring Cost on the Large-Scale Architecture of Human Cortical Connectivity

**DOI:** 10.1371/journal.pcbi.1003557

**Published:** 2014-04-03

**Authors:** David Samu, Anil K. Seth, Thomas Nowotny

**Affiliations:** 1 Sussex Neuroscience, CCNR, Informatics, University of Sussex, Falmer, Brighton, United Kingdom; 2 Sackler Centre for Consciousness Science, Informatics, University of Sussex, Falmer, Brighton, United Kingdom; Indiana University, United States of America

## Abstract

In the past two decades some fundamental properties of cortical connectivity have been discovered: small-world structure, pronounced hierarchical and modular organisation, and strong core and rich-club structures. A common assumption when interpreting results of this kind is that the observed structural properties are present to enable the brain's function. However, the brain is also embedded into the limited space of the skull and its wiring has associated developmental and metabolic costs. These basic physical and economic aspects place separate, often conflicting, constraints on the brain's connectivity, which must be characterized in order to understand the true relationship between brain structure and function. To address this challenge, here we ask which, and to what extent, aspects of the structural organisation of the brain are conserved if we preserve specific spatial and topological properties of the brain but otherwise randomise its connectivity. We perform a comparative analysis of a connectivity map of the cortical connectome both on high- and low-resolutions utilising three different types of surrogate networks: spatially unconstrained (‘random’), connection length preserving (‘spatial’), and connection length optimised (‘reduced’) surrogates. We find that unconstrained randomisation markedly diminishes all investigated architectural properties of cortical connectivity. By contrast, spatial and reduced surrogates largely preserve most properties and, interestingly, often more so in the reduced surrogates. Specifically, our results suggest that the cortical network is less tightly integrated than its spatial constraints would allow, but more strongly segregated than its spatial constraints would necessitate. We additionally find that hierarchical organisation and rich-club structure of the cortical connectivity are largely preserved in spatial and reduced surrogates and hence may be partially attributable to cortical wiring constraints. In contrast, the high modularity and strong s-core of the high-resolution cortical network are significantly stronger than in the surrogates, underlining their potential functional relevance in the brain.

## Author Summary

Macroscopic regions in the grey matter of the human brain are intricately connected by white-matter pathways, forming the extremely complex network of the brain. Analysing this brain network may provide us insights on how anatomy enables brain function and, ultimately, cognition and consciousness. Various important principles of organization have indeed been consistently identified in the brain's structural connectivity, such as a small-world and modular architecture. However, it is currently unclear which of these principles are functionally relevant, and which are merely the consequence of more basic constraints of the brain, such as its three-dimensional spatial embedding into the limited volume of the skull or the high metabolic cost of long-range connections. In this paper, we model what aspects of the structural organization of the brain are affected by its wiring constraints by assessing how far these aspects are preserved in brain-like networks with varying spatial wiring constraints. We find that all investigated features of brain organization also appear in spatially constrained networks, but we also discover that several of the features are more pronounced in the brain than its wiring constraints alone would necessitate. These findings suggest the functional relevance of the ‘over-expressed’ properties of brain architecture.

## Introduction

The physical brain is a network of extraordinary complexity on multiple spatial scales. On the macroscopic scale, regions are connected by a large number of white-matter projections that form an intricate system: the connectome [1]. Understanding the principles of the large-scale architecture of the brain, how this architecture shapes brain dynamics to in turn support brain function and human behaviour, is a central challenge for contemporary neuroscience [2], [3].

Recent advances in non-invasive anatomical [4]–[6] and functional [7] imaging techniques, along with the development of automated, high throughput post-processing methods [8] now allow the application of complex network science as a principled and systematic framework for studying the connectome [2], [3]. Accordingly, numerous principles of organisation in the large-scale structural anatomy of the brain have been characterized, including small-world properties [9], hierarchical architecture [10], modular structure [11], the existence of a strong structural core [12] and a so-called ‘rich-club’ organisation [13]. Exposing both the structural origin and functional relevance of these properties of the human connectome is an essential, but difficult step towards a deeper understanding of the large-scale organisation of the brain.

A common approach to evaluating the significance of a particular network property, observed in a particular network, is via surrogate or null-hypothesis comparison [14], [15]. In this approach, a set of surrogate networks represents a null-hypothesis for the target network property by preserving some *a priori* chosen properties of the network under investigation, while randomizing other network properties. Quantitative comparison of the original network with the ensemble of surrogate networks allows drawing conclusions on the significance of the target property of the network with respect to those properties preserved in the ensemble. Therefore, in its essence, surrogate network comparison allows testing if some, usually very elementary, properties of the target network induce, or at least contribute to, the expression of some of its more global and complex network properties.

When choosing appropriate surrogate networks, the most widely used null-hypothesis properties are size (number of nodes), connection density (number of edges) and degree distribution (the number of connections of each node). This approach – which we term the ‘random surrogate’ approach – has illuminated the topological investigation of many abstract, spatially-unembedded networks, including the World Wide Web, semantic networks, food-webs, and gene-regulatory and metabolic networks [14], [16]. It is also routinely applied in the analysis of brain networks in order to demonstrate that global, ‘higher order’ network property of brain maps, such as modularity or ‘small-worldness’, cannot be attributed solely to these basic network properties [10], [11], [17].

Physical networks like the brain are, however, embedded into physical space and are therefore subject to additional constraints deriving from the costs of developing and maintaining connections [18] which are not conserved by random surrogates. Random surrogates therefore represent a rather loosely constrained null-hypothesis set for physical networks. Specifically, they tend to possess a large number of long-range connections because they ‘smooth’ local inhomogeneities of physical networks. They thus form highly and rather homogeneously integrated networks, while at the same time lacking the high topological segregation (locally dense, globally sparse inter-connectivity) associated with predominantly local connectivity, which is one of the most prominent features of brain networks [19]. When compared against random surrogates, then, certain properties of brain networks may appear to be highly distinctive even though they can be attributed to the spatial constraints of its embedding into the physical world (wiring cost) and/or of the economic pressure of minimising the number of the energetically expensive long-range connections (metabolic cost) [18].

To address this problem, so-called ‘lattice surrogates’ have been introduced [15], [20]–[22] to preserve (or rather increase) the high segregation of brain networks. The motivation behind lattice surrogates, originating from the Watts–Strogatz notion of ‘small-worldness’ [23], was to represent a lattice-like, topologically over-segregated (and thus under-integrated) surrogate network type, the opposite of random surrogates in a sense, and to compare the target network with these two extremes. This is reflected in the rule commonly used to generate lattice surrogates from the connectivity of a brain network (during a ‘random’ network rewiring process, edge swaps are only made if the nonzero entries of the resulting connectivity matrix are located closer to the main diagonal [15], [20]), which is only indirectly linked to physical distance through some arbitrary spatial ordering of the network nodes. For this reason, lattice surrogates are only partially appropriate as a null-hypothesis network set for physical wiring constraints of brain networks. Furthermore, lattice surrogates are designed to reduce, rather than preserve, network connection lengths thus further undermining their utility in assessing the effects of wiring constraints on cortical network properties.

In this paper, we introduce two new classes of surrogates, *spatial surrogates* and *reduced surrogates*, Like random surrogates, spatial surrogates preserve network size, connection density, and degree distribution, but (unlike random surrogates) they also preserve the wiring length distribution of the target network. Reduced surrogates are like spatial surrogates with the difference that they do not preserve but actually reduce overall network wiring, in similar way to traditional lattice surrogates, but in a spatially well-defined and controlled manner. We reasoned that in virtue of these properties, these surrogates provide improved baselines by which to assess the extent to which a target network property can be attributed to cortical wiring constraints [18].

**Figure 1:**
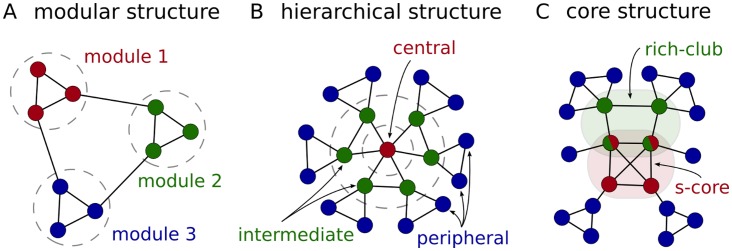
Illustration of network organisation principles. (A) Modular structure composed of a set of highly intra-connected but sparsely inter-connected group of regions (modules, clusters or communities). (B) A three level hierarchical structure composed of a central (high degree – low clustering), an intermediate (medium degree – medium clustering) and a peripheral layer (low degree – high clustering). (C) Two core structures of a network: rich-club (highest degree regions) and s-core (most densely intra-connected regions). Note that while the two structures are not necessarily equivalent, they are likely to possess significant overlap.

This approach enables us to evaluate a number of prominent findings regarding the structural properties of the connectome (see Figure 1) with respect to the extent to which these properties are preserved in the novel spatial surrogates as compared to random and connection length optimised (reduced) surrogates. To ensure robustness we perform these analyses on both weighted and unweighted (binary), and on the full resolution (998 regions) as well as on a lowered resolution (66 regions) version of the cortical structural connectivity data set provided by Hagmann et al. [12]. Overall, the method allows us to distinguish those significant network properties of the connectome that are derivable from its predominantly local, spatially segregated connectivity (as indicated when both the cortical network and its spatial and reduced surrogates differ from random surrogates) from those that are the consequences of some other, primarily not (or not only) spatial, but potentially more functionally relevant organisation principle of cortical connectivity (as indicated when the cortical network differs from all of its surrogate groups).

Specifically, during the evaluation of each specific network property, the logic of our surrogate analysis is the following (see Table 1). We measure the expression of the network property in the cortical network and every surrogate group by an appropriate complex network metric. If all surrogate groups exhibit similar metric indices to that of the cortex, then the basic network properties preserved in all surrogates (the number of regions, number of white-matter projections and regional degree distribution of the cortical network) appear to be sufficient for the observed expression of the investigated network property. If, however, all spatially constrained networks (cortical network, spatial and reduced surrogates) exhibit similar values, but differ from random surrogates, we reason that cortical wiring constraints may account for the level of expression of that network property in the cortical network. Additionally, if the cortical network is more similar to spatial than to reduced surrogates, we reason that solely the presence of long-range connections in the cortex may facilitate the network property, irrespectively of the specific arrangement of these connections in the cortex. If, however, the cortical network is more similar to reduced than to spatial surrogates, then we reason that the predominantly local (short-range) connectivity of the cortex can account for the expression of the network property even in the absence of long-range cortical connections (as indicated by the similarity between the cortical network and reduced surrogates). In addition, this case also indicates that the particular arrangement of long-range cortical connections appears to be such that it does not interfere with (strengthen or hinder) the expression of the network property (as indicated by spatial surrogates, with randomised long-range connections, being different from both the cortical network and reduced surrogates). Finally, if the cortical network differs from every surrogate ensemble, we reason that the network property is specific to the particular connectivity of the cortex, it cannot fully be attributed to the topological properties and wiring constraints that are conserved in the surrogates, but instead may be a more functionally relevant organisation feature of cortical connectivity.

**Table 1:** Interpretation of analysis results for a generic network property for each configuration of relative metric indices between the cortical network and its surrogates.

Relations between cortical and surrogate measure indices	Interpretation
M_C_≈M_R_≈M_S_≈M_rnd_	P is present equally in all surrogates and in the target network. Its expression can therefore be attributed to basic network properties, namely the number of regions, number of white-matter projections and regional degree distribution of the cortical network.
M_C_≈M_S_≈M_R_≉M_rnd_	P may largely be attributed to cortical wiring constraints alone.
M_C_≈M_S_≉M_R_≉M_rnd_	P may largely be attributed to cortical wiring constraints and to the mere presence of sparse long-range cortical connections, irrespectively of their specific arrangement.
M_C_≈M_R_≉M_S_≉M_rnd_	P may largely be attributed to cortical wiring constraints (M_C_≈M_R_) and to the specific arrangement of long-range cortical connections which do not interfere with (strengthen or hinder) the expression of P (M_C_≉M_S_).
M_C_≉M_R_≉M_S_≉M_rnd_	P cannot be fully accounted for in terms of basic topological cortical properties and cortical wiring constraints, and hence determined by other evolutionary pressures, maybe because it is more functionally relevant.

P: a generic network property; M: a complex network metric measuring the expression of P; M_C_: metric value of cortical network; M_R_, M_S_, M_rnd_: mean metric value of reduced, spatial and random surrogates, respectively.

## Methods

### Cortical connectivity dataset

**Figure 2:**
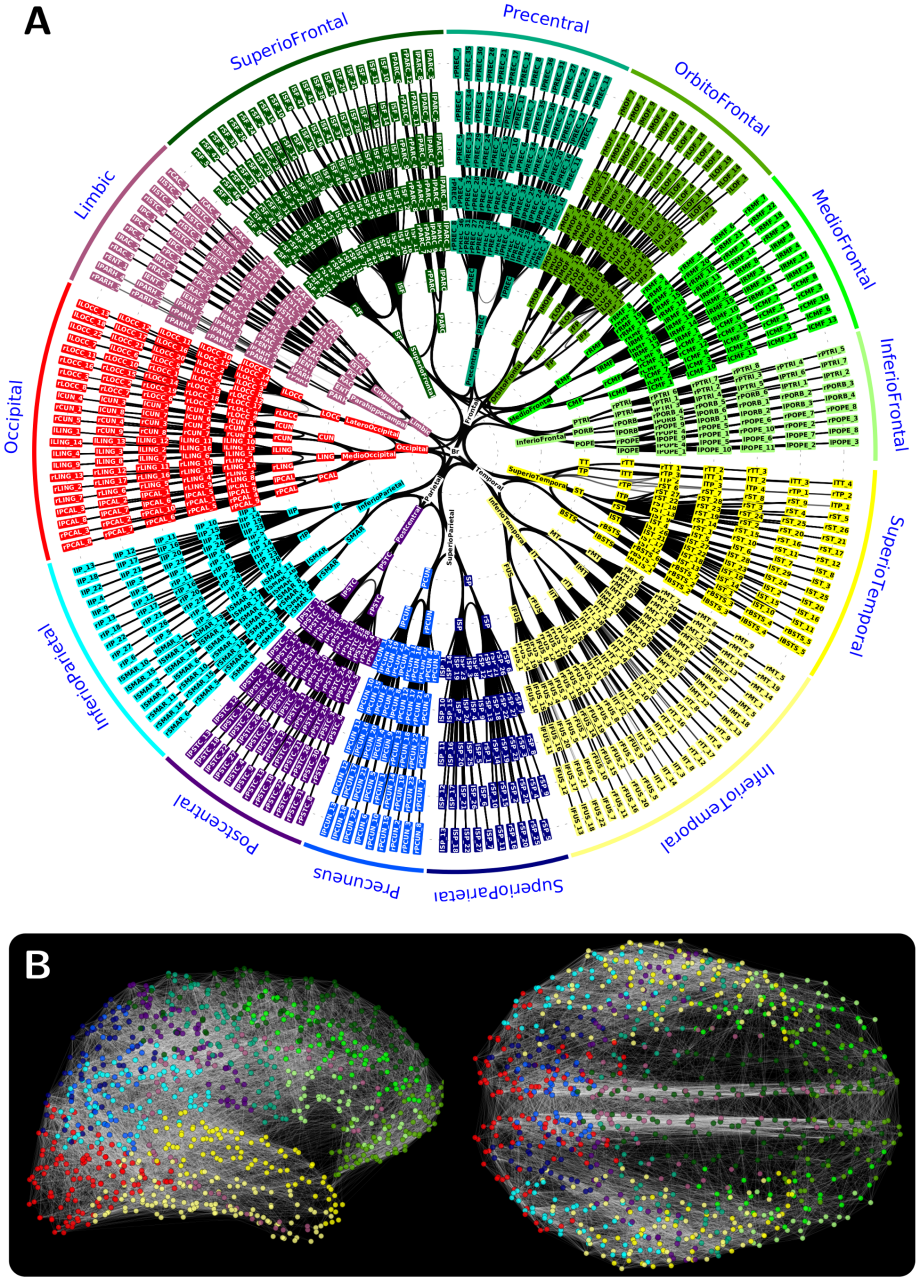
Structural connectivity dataset. Visualization of the structural connectivity dataset [12] used in this study, illustrated using an abstract radial layout (A), and on coronal and horizontal projections (B). In (A) the 998 considered cortical regions are arranged on the five outermost circles and grouped by main anatomical structures (see sectors at perimeter). Black curved lines illustrate connections between cortical regions, ‘bundled’ together along the shared portion of their paths in the abstract layout (for a general introduction of the layout, see [97]). In (B) the brain regions are shown in coronal and horizontal projections of the cortical anatomy, colour coded as in (A) according to large anatomical structures.

We use the cortical connectivity network of Hagmann et al. [12] (Figure 2). This data was obtained by non-invasive tracing of white-matter projections linking pairs of cortical sites in the brains of five human subjects, combining magnetic resonance imaging (MRI) and diffusion spectrum imaging (DSI) techniques, semi-automated brain parcellation, diffusion tractography and appropriate post-processing methods. The individual connectivity networks of the five subjects were aggregated into a single network in order to reduce the impact of inter-subject variability. The resulting dataset is a compact network representation of cortical grey matter regions as network nodes, and their connecting white-matter fibre bundles as edges. For a detailed description of the acquisition procedure and validation test results of the procedure, see the original paper and [8].

By the nature of its processing pipeline, the network consists of a two-level hierarchical parcellation of the cortex: it is composed of 66 anatomical regions at the higher level, and of 998 regions of interest (ROIs) at the lower level. Each node on the level of ROIs represents an area of the cortical surface of approximately 1.5 cm^2^ size (region), and there are a total of 17,865 undirected weighted connections between these regions. These figures result in a fairly sparse, 3.6% connection density network on the high-resolution cortical parcellation (i.e., on the lower hierarchical level of the segmentation). For the low-resolution network, similarly to [23], we calculate the strength of the connection between every two-region pair by summing the weights of all the high-resolution connections linking the ROIs that compose the two cortical regions. This method results in 574 aggregated white-matter fibre bundles between the 66 regions on the low-resolution parcellation, which increases the connection density of the low-resolution cortical connectivity to 26.8%.

While a few studies on high-resolution structural connectivity networks have appeared recently [e.g.], [ 12], [24,25], many earlier results, in particular those based on the data set used here, have relied on low-resolution data [e.g.], [ 26], [27,28]. Although focussing on low-resolution data allows comparing to earlier low-resolution studies on other brain networks [e.g.], [ 11,20], utilizing the information afforded by the available higher resolution connectivity may influence the outcome of complex network analysis [29], [30] and has the benefit of maximizing usage of the available information. Here, we primarily analyse the high-resolution, 998-node anatomical connectivity network (see Figure 2), but we also compare to lower resolution results where appropriate.

### Surrogate network generation

We employ three types of null-hypothesis networks, namely *random*, *spatial* and *reduced* surrogate networks. All three surrogate types preserve the size (number of nodes), connection density (number of edges) and degree distribution (the number of connections of each node) of the cortical network, and differ from each other only in their physical wiring constraints: random surrogates are spatially non-constrained, spatial surrogates preserve the total wiring length of the cortical regions (and thus that of the entire cortical network globally), and reduced surrogates possess reduced wiring lengths.

All three types of surrogate networks were generated by the widely applied iterative rewiring algorithm [14], [31], the basic version of which proceeds as follows: Starting from the original cortical network, in each iteration two connections, (*r_1_*, *r_2_*) and (*r_3_*, *r_4_*), are randomly chosen (where *r_i_* refers to region *i*). After ensuring that no self-connections or parallel links (multiple connections between two regions) would be created, the two original connections are swapped to (*r_1_*, *r_3_*) and (*r_2_*, *r_4_*).

The above basic rewiring algorithm is sufficient to generate random surrogate networks. For the spatially constrained surrogate network sets, we incorporated the following additional rewiring conditions: each rewiring step is only executed if the resulting total connection length of every region (i) does not exceed that of the region in the original cortical network (for spatial surrogates), or (ii) is reduced in every step (for reduced surrogates). Because the complex curving trajectories of pathways cannot be preserved during rewiring, connection lengths are approximated by Euclidean distances between the positions of the region-pairs, for both cortical and surrogate networks. In the case of random and spatial surrogates, the procedure is terminated when each connection has been rewired 20 times on average (20 * n_e_/2 = 178650 connection swaps). For the most constrained reduced surrogates this stopping criterion is too severe because, as the algorithm progresses, progressively fewer rewiring operations with connection length reductions can be found. As a compromise, for this surrogate we chose to rewire each connection only once on average (n_e_/2 = 8932 connection swaps), resulting in a reasonably diverse (i.e., not overly self-similar) and yet well-optimised set of reduced surrogate networks (see *Results*). On both resolutions, we generated n = 20 networks for all three surrogate types.

### Assessing topological similarity

To assess the topological similarity between the cortical connectivity network and its surrogates, we calculated the overlap between the set of connections of the cortical network and the surrogate networks, both in binary and weighted fashion. Specifically, we calculated the binary and weighted overlap between the cortical network C and each of its surrogate S using a modified version of the Sørensen similarity quotient QS [32], which measures the similarity or relative overlap between two sets by the quotient of their intersection and union. We define the binary version of the similarity measure QS^b^ as:
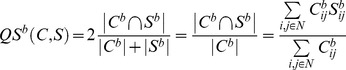
(1)where N is the (identical) set of all nodes in networks C and S, and C^b^ (S^b^) is the binarized connectivity matrix of C (S) with C^b^_ij_ (S^b^_ij_) being 1 if there is a link between nodes *i* and *j* in C (S) and 0 otherwise. Note that the number of connections in C and S, |C^b^| and |S^b^|, are equal, and that the product C^b^_ij_ S^b^_ij_ is 1 if there is a connection between node *i* and *j* both in C and S, and 0 otherwise.

Similarly, we define the *weighted* similarity quotient QS^w^ as:
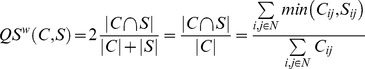
(2)where C_ij_ and S_ij_ are the connection weights between regions *i* and *j* in networks C and S, respectively (0 if the two regions are not connected).

QS^b^ and QS^w^ measure the relative similarity between the connection sets of two networks C and S that are defined on the same set of regions. Both QS^b^ and QS^w^ are normalised similarity quotients taking the value 0 if the two networks share no common connection (minimal similarity), and 1 if the networks are equivalent, that is, they are composed of exactly the same set of binary/weighted connections (maximal similarity). We use both measures because they assess network similarity of two networks in a complementary manner: the overlap in the binary layout of the two networks can only be assessed faithfully by QS^b^ (if the networks are different only in a small number of very high weight links, QS^w^ is already low, despite the high binary overlap), while QS^w^ accounts for the importance (weight) of the connections (if the networks are different only in a number of very low weight links, QS^b^ is lower, despite the high weighted overlap).

### Assessing spatial similarity

We assess *spatial* network similarity between a network and its surrogates as the average spatial replacement of the connections of each region *r*, that is, the average change in the positions of all topologically adjacent (linked) regions of *r* (its topological neighbourhood) in the original and surrogate networks. The theoretically optimal solution for measuring such spatial displacement of the connections would require finding the ‘best matching pairing’ between the original and the rewired neighbour sets of *r*, i.e., the pairing in which the sum of distances between the (original, rewired) region-pairs is minimal. An exhaustive search for this optimal pairing is however computationally infeasible (given that the regions on average possess 35 connections, a lower estimate on the average number of pairings to check per region is 35!≈10^40^), therefore we developed and utilized the following algorithm to find an approximation of the optimal pairing.

Given the set of the original topological neighbours of region *r* in the cortical connectivity, L = [l_1_, l_2_, …], and the set of *r*'s rewired neighbours in the surrogate network, M = [m_1_, m_2_, …], we calculate the pair-wise distances D(L,M) = [d(l_1_,m_1_), d(l_1_,m_2_), …, d(l_2_,m_1_), d(l_2_,m_2_), …] between all element-pairs of the two sets. Then we sort D(L,M) ascending (from the closest to the farthest original-rewired neighbour pairs), and, while iteratively going through the region-pairs of this sorted list, we put the current (*l_i_*, *m_j_*) pair into pairing list P if and only if neither *l_i_* nor *m_j_* is currently in P.

Although the resultant pairing P provided by the ‘greedy algorithm’ above is not guaranteed to be the optimal pairing P_opt_ between L and M, i.e., the one having the lowest sum of (original, rewired) pair-wise distances, it is expected to provide a reasonable estimate on P_opt_ given the close to homogeneous spatial distribution of the regions of the cortical network on the spheroid surface of the cortex [12].

Having obtained P for every cortical region *r*, we calculate the global relative spatial displacement D between the cortical connectivity C and its surrogate network S as:
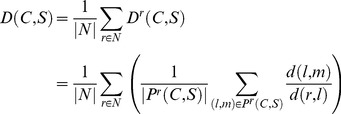
(3)where N is the set of all regions in the networks (identical in C and S), D^r^(C,S) is the average displacement of *r*'s neighbours in C and S, P^r^(C,S) contains the (original, rewired) neighbour-pairs of *r* for C and S, and *d(a,b)* is the spatial distance between regions *a* and *b*. With the above definition, D measures the distances between the original and the rewired neighbours of *r* (connection displacement) normalised by the distance of the original (cortical) neighbour from *r*, averaged over all connections and all cortical regions. D = 0 if there is no spatial displacement between the two networks, meaning that they are (both topologically and spatially) identical. A low D value indicates that there is only minor spatial displacement in the neighbour sets of the regions on average, while higher D values indicate a greater neighbourhood displacement, hence a larger difference in the spatial layout between the cortical connectivity and its surrogate network. Generally, the upper limit of D depends on the particular spatial distribution of the nodes and edges of the original network as well as of the wiring constraints of the rewired network in a complex manner. As a simplifying rule for the sparsely and predominantly locally connected (high-resolution) cortical network, however, we can regard D values on the order of 1 as indicators of substantial spatial neighbourhood displacement.

### Global efficiency

A basic measure of network integration, global network efficiency [33] is the average of the inverse of the shortest path lengths *d_ij_* between a node *i* and every other network node *j*, averaged over all network nodes:
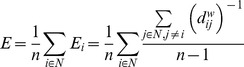
(4)where E_i_ is the efficiency of node *i*, *n* is the number of nodes, and *d^w^_ij_* is the weighted shortest path length between nodes *i* and *j* (the minimal of the weighted sums of constituent edges along each path between *i* and *j*, where connection weights are the reciprocal of their strength). High global efficiency implies that, on average, nodes require fewer intermediate steps along stronger (higher weight) edges to reach other nodes; therefore, networks with higher global efficiency possess greater potential for efficient internal information exchange and integration. The advantage of efficiency as a measure for integration over the more traditional measure of the mean shortest path length [15] is that efficiency can be computed for networks with multiple components, and generally is a more balanced measure due to the fact that the mean shortest path length can be strongly biased by the presence of only a few, very long paths [34].

### Clustering coefficient

A basic metric of network segregation, the clustering coefficient [23] is the fraction of triangles around a node (the proportion of the node's topological neighbour pairs that are connected with each other), averaged over all network nodes. The *weighted* clustering coefficient [35], which we use in this study on weighted networks, is defined as follows:
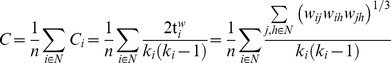
(5)where C_i_ is the clustering coefficient of node *i*, *k_i_* is the degree of *i*, *t_i_^w^* is the (weighted) geometric mean of triangles around *i*, *w_ij_* is the (normalised) connection weight between regions *i* and *j* (0 if *i* and *j* are not linked). The clustering coefficient of a node is high (1) if many (all) of its neighbours are also directly connected pair-wise (by strength 1 connections in the weighted version of the measure), and it is 0 if none of its neighbour-pairs are directly connected. The clustering coefficient hence measures the (topologically) local density of connectivity of a network.

### Small-world index

Informally, a small-world network is a highly segregated (i.e., preferentially locally connected) and yet relatively highly integrated (i.e., easily traversable) network [23]. For the quantitative assessment of small-worldness, the network's high integration is usually translated to relatively short path lengths, while strong segregation is measured by a high level of clustering [36]. Among the several formulae developed to assess the degree of small-worldness of complex networks (e.g. [33], [37]), we chose an altered version of the Humphries–Gurney small-worldness index [37], modified in the following way:
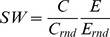
(6)where C and C_rnd_ are the clustering coefficient of the network and its random surrogates, while E and E_rnd_ are their global efficiencies, respectively [15]. We note that Humphries and Gurney in [37] use average shortest path lengths instead of efficiency; however we prefer efficiency for the reasons stated above. A network is then said to be small-world if its clustering coefficient is larger than those of its random surrogates (C≫C_rnd_), while their efficiencies (shortest path lengths) are comparable (E≈E_rnd_), resulting in SW≫1 [37].

### Measuring network hierarchy

Using the intuition that high degree nodes should occupy a topologically central position in a hierarchical network as a starting point, Ravasz and Barabási introduced the simple but elegant hierarchy coefficient β for assessing hierarchical architecture in scale-free networks [38]. Noticing a distinctively exponential relationship between node degrees and clustering coefficients for various synthetic and real-world scale-free networks, they proposed that the exponent β of this relationship quantifies the tendency of high degree nodes to be linked to a large but sparsely intra-connected neighbour set (hence exhibiting low clustering) and thus effectively serving as connector nodes between segregated parts of the network [38].

Unfortunately, the human cortical network under study, and therefore also its degree-distribution preserving surrogates, exhibit an exponential, rather than scale-free degree distribution [12], and the node degree – clustering relationship does not show a clear exponential shape, so that the β index of Ravasz and Barabási [38] cannot be applied directly. However, their basic idea remains valid irrespective of the specific shape of the functional degree to clustering relationship. Therefore, we here characterize hierarchical organisation by directly observing the degree to clustering relationship in the cortical network and in its surrogates. Specifically, in sparsely connected and locally highly clustered networks, (of the sort studied here, see *Results*), high degree nodes of a network that possess a lower than average clustering coefficient are typically in a position to connect segregated parts of the network, suggesting a hierarchical element of the architecture with these high degree nodes in its centre (see Figure 1B). In contrast, equal or higher than average clustering coefficients of high degree nodes indicate more homogeneous architectures and the lack of the hierarchical organisation pattern investigated in [38]. We note that the specific kind of topological organisation described above is of course not the only conceivable network architecture that exhibits hierarchical attributes. It is nevertheless the one that has previously been discovered in many sparsely connected, but highly clustered and modular real-world networks [38], making it a good candidate to test for here.

### Module partition detection

The modularity index Q, proposed by Newman [39], has proved to be a highly accurate and powerful indicator of the modularity strength of a given partitioning of a complex network [16], [40]. Given a set of node groups (modules or communities) M, that fully partition the network without overlaps, the modularity index Q of that partition is given by
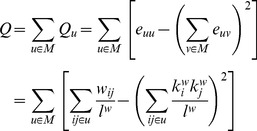
(7)where Q_u_ is the modularity index of module *u*, *e_uv_* is the proportion of all weighted edges *w_ij_* between modules *u* and *v* in the network, *l^w^* is the sum of all weights in the network, and *k_i_^w^* is the sum of all connection weights of node *i*.

Numerous algorithms have been developed to recover the modular structure of complex networks utilising Q as a ‘fitness’ measure to be optimised by some means (for reviews, see [16], [40]). In this study, we use the simple and elegant spectral algorithm developed by Newman [41]. Starting from the entire network as a single module, this algorithm iteratively splits each module into two, at each step finding the optimal bipartition by utilising a so-called ‘modularity matrix’ derived from the network's connectivity matrix. The leading eigenvector of the modularity matrix determines the node composition of the two sub-modules of each module to be split. The algorithm stops when no more increase in the global modularity index Q can be achieved by any additional split [41]. Along with its high accuracy, Newman's module detection procedure has the additional advantages of being a divisive, deterministic and generalisable method with low computational cost. See [41] for a detailed description of its implementation.

### Consistency of module partitions

We measured the consistency of the cortical module partition in surrogate networks with the *scaled inclusivity index* [42]. Application of this measure capitalized on the fact that the cortical network and its surrogates are defined on the same set of nodes (cortical regions) and differ only in their connection sets. Additionally, scaled inclusivity has the advantage of making no assumptions on the investigated partitions, and is thus generically applicable even on partition-pairs which differ in the number and sizes of modules they contain. For other pair-counting, cluster-matching, and information-theoretic techniques applied to compare module (community) structures of different networks, see [43]–[45].

The calculation of the scaled inclusivity index proceeded as follows. First, the individual module partitions of the cortical and surrogate networks were identified independently by Newman's spectral algorithm (introduced above). Then, the cortical module partition Q^C^, composed of *m* modules, was taken as a reference partition, and its match with the partition Q^Si^ of each network *i* of surrogate group S, composed of *n* modules, was assessed by calculating the *n*×*m* module-by-module similarity matrix X^iC^, which (*p*,*q*)-th element is calculated as:
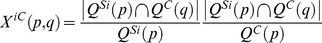
(8)where Q^Si^(p) is the set of nodes (regions) belonging to the *p*-th module in Q^Si^ and Q^C^(q) is the set of nodes belonging to the *q*-th module in Q^C^. The resulting values range from 0 to 1, where X^iC^(p,q) = 0 indicates zero overlap between the modules *p* and *q* (i.e., they do not share any node), and 1 indicates that the two modules are identical (i.e., they are composed of the same set of nodes).

After calculating the matrix X for all networks in a surrogate group, the scaled inclusivity index SI of each cortical region is calculated as the mean of the similarity indices X^iC^(p,q) between all modules Q^Si^(p) and Q^C^(q) that contain the region, averaged over all surrogate networks *i*. Thus, scaled inclusivity measures how consistently a region is classified in each surrogate group, based on how well its cortical modules match with its surrogate modules, on average. We stress that SI is intended as a generically applicable metric to measure the *degree of similarity between the module classification of network nodes*, and it does not aim to accurately measure the actual magnitude of ‘overlap’ between the partitions (see [42] and Eq. 8 above).

### K-core/s-core detection

The ‘core’ of a network is usually determined by an iterative peeling algorithm. These algorithms, at each step, remove (‘peel off’) a set of ‘shell’ or ‘crust’ nodes, in order to progressively focus on the more ‘centralised’ nodes. Centralisation in these procedures is assessed by a specific ‘coreness condition’, as described below.

To find the core structures of binary and weighted networks, we used the k-core and s-core decomposition methods, respectively. The *k-core* of the network [46], for a given degree *k*, is the maximal set of nodes that are connected to at least *k* other nodes in the core. The k-coreness index of a node is then the highest degree *k* for which the node is still a member of the k-core. Similarly, the weighted variant of the k-core, the *s-core* of the network [12] is the group of nodes in which each node has a summed connection strength of at least *s* towards the rest of the s-core (i.e., the sum of the weights of its intra-core connections is not less than *s*). For increasing *s* (*k*), the s-core (k-core) shrinks progressively and the tightest or innermost s-core (k-core) of the network [simply s-core (k-core) from here on] is the set of remaining nodes in the last non-empty s-core (k-core).

### Rich-clubness assessment

The so-called rich-club phenomenon is the tendency of high degree nodes to be preferentially connected to each other [47], [48]. The degree of ‘rich-clubness’ is usually measured by the k-density function φ(k) of the network, which is the internal connection density among all nodes with degree larger than k. There is a basic difference between k-core/s-core and rich-club properties: while k-core and s-core nodes are selected by their connections *within* the subnetwork formed by the core, rich-club nodes are chosen simply and solely on the basis of their *global* degree in the entire network. (Of course the ‘rich-clubness’ of this subnetwork does then depend on its internal connectivity.)

A possible weighted variant of the rich-club measure, as introduced in [49], evaluates the tendency of the highest connection weights to be distributed among high degree (‘rich’) nodes. However, this variant, due to normalisation by the number of edges, is a connection density-independent index of *weight* centralisation and thus loses the ability of the unweighted rich-club index to measure *edge* centralisation among high degree nodes. Here we propose a novel weighted version of rich-clubness, which is sensitive to both properties, connection density and weight centralisation, and may hence be a more appropriate generalisation of the unweighted rich-club index to weighted networks.

We define *weighted rich-clubness* as the internal weighted connection density φ^w^(k) of the set of nodes with degrees larger than k, N_>k_, which is the ratio between the sum of connection weights W_>k_ among the nodes in N_>k_ and the maximum of their possible weight sum, W^max^_>k_:
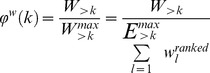
(9)where 
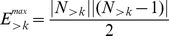
 is the maximum possible number of edges among the nodes in N_>k_, and w^ranked^_l_ is the weight of the *l* ^th^ strongest (highest weight) edges in the network.

φ^w^(k) defines a normalised measure of coreness, which takes a value in [0,1] for each degree k. φ^w^(k) is 1 only in the extreme case where N_>k_ is fully connected by exactly the strongest connections of the network. In general, φ^w^(k) measures the fraction of total interconnection strength within N_>k_ relative to this theoretical maximum (as defined by the connection weights present in the network).

Note that in Eq. 9 the denominator is not calculable if E^max^_>k_ is greater than the number of edges, E, in the network. This condition renders the interpretation domain of φ^w^ dependent on the connection density of the investigated network, implying that φ^w^ is meaningful for weighted rich-clubness measurements only for that fraction of the highest degree nodes N_>kmin_. Specifically, for undirected graphs, the number of these nodes |N_>kmin_| cannot be larger than the real solution of the quadratic equation
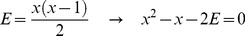
(10)
Eq. 10 specifies the largest number of nodes *x* that can still be fully interconnected by the existing number of edges E in the network. The cortical network under study has E = 17865 connections, hence we obtain |N_>kmin_| = 188 nodes as the largest weighted rich-club size that can be assessed by our measure. This corresponds to 18.8% of the nodes of the entire network, and gives φ^w^ (k) the domain of k∈[k_min_, k_max_], where k_max_ = 97 is the largest node degree in the network, and k_min_ = 49 is the degree of the 188th node in the degree rank ordered node list. We note that, apart from of this interpretation limit of the measure, when applied to unweighted (binary) networks φ^w^ gives the same result as the traditional rich-club metric, underlining that φ^w^ can be interpreted as a generalisation of this traditional measure for weighted networks.

### Degree assortativity

Degree assortativity is a global measure of the tendency of nodes to be preferentially connected to other nodes with similar degree [50]. Degree assortativity is thus closely related to the phenomenon of rich-club formation, although while the latter only accounts for high degree nodes, the former measures preferential connectedness across nodes of all degrees. The assortativity coefficient *r* of a network is formally defined as:
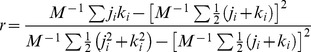
(11)where *j_i_*, *k_i_* are the degrees of the nod es at the ends of edge *i*, and M is the number of edges [50]. Degree assortativity is a normalised measure (−1≤*r*≤1), so that a network has positive *r* assortativity values if its edges tend to connect nodes of similar degree, while negative assortativity values indicate the tendency for nodes with different degrees to be linked. A network with *r*≈0 expresses neither of these trends, and is non-assortative.

## Results

In the analyses presented below we used the structural connectivity network of the human cortex obtained by Hagmann et al. [12] comprising 998 regions of interest and 17,865 undirected and weighted connections (Figure 2), see *Methods*. Unlike many previous studies [e.g.], [ 26], [27,28], we performed analysis on both the full maximal resolution and on a low-resolution sub-sampling of the data set and surrogate networks of the same size, and on both weighted and unweighted (binary) versions of these networks. Additionally, we repeated the analysis on a single cortical hemisphere of the high-resolution network, in order to test for any artefacts arising from the features of inter-hemispheric connections (see *Single hemisphere analysis*). In the following, we present the results with a focus on the high-resolution weighted connectivity type (as it contains the most information), and discuss the findings on the other network types at the end of the section (*Results on low-resolution and binary connectivity types*). In the following we first describe validation of the three surrogate sets. We then compare standard topological integration and segregation properties of cortical and surrogate networks, and finally report analysis of more complex network properties such as small-worldness, hierarchy, modularity and core formation.

### Validation of surrogate networks: Topological similarity

**Figure 3:**
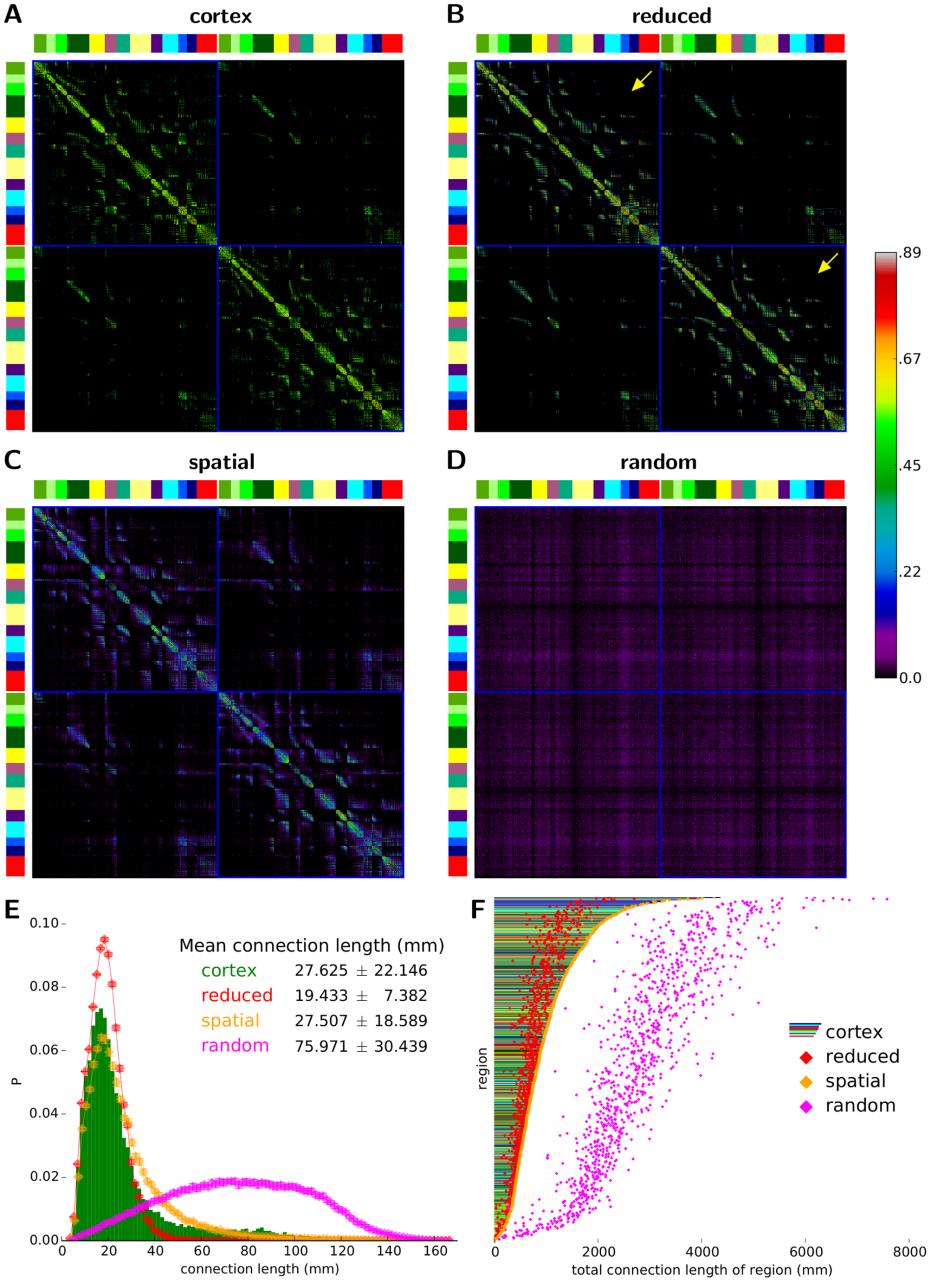
Connectivity matrices and spatial properties of high-resolution cortical connectivity and its surrogate networks. (A) Connectivity matrix of high-resolution cortical connectivity. (B–D) Averaged connection matrices (projection strength weighted frequencies of connection occurrences) of the three surrogate groups (‘reduced’, ‘spatial’ and ‘random’). See colour-bar on right for scale of all four matrices (A–D). All matrices are symmetric due to the undirected nature of the networks. Cortical regions are ordered first by hemisphere (top left sub-square is left, bottom right is right hemisphere), then by containing anatomical structures (left and top colour stripes, for structural colour-code, see sector names in Figure 2A), finally by spatial positions along the rostro-caudal axis. Note that within each hemisphere (top left and bottom right sub-squares) reduced surrogates have fewer connections at areas farther away from the main diagonal than the cortical network does (see yellow arrows in B), due to them having preferentially lost long-range connections. (E) Connection length (region-region distance) distribution of cortical network (bars) and its surrogates (diamonds), with mean +/− standard deviation indicated in the legend. Whiskers on diamonds denote the standard deviation between networks within each surrogate group. Note the highly similar, but somewhat shorter tailed spatial surrogate distribution, and the significantly narrower and shorter tailed reduced surrogate distribution, reflected in their lowered standard deviations (see figure legend) (F) Histogram of total connection length of regions (sum of distances from all neighbours). Each row corresponds to a single region, bars represent values in the cortical network and are coloured to indicate the corresponding anatomical structure (see Figure 2B). Diamonds denote mean of total connection lengths of region within each surrogate groups (see legend). Note that total connection lengths of regions is preserved in spatial surrogates, while they have been decreased in reduced surrogates and increased in random surrogates.

The high-resolution weighted cortical connectivity matrix and averaged connectivity matrices of the three surrogate sets are illustrated in Figure 3A–D. To allow meaningful comparisons, surrogate networks need to be sufficiently randomised. The rewiring algorithms, as outlined in *Methods*, are constrained by several factors during the randomisation of cortical connectivity. In order to assess that sufficient randomisation has been achieved in spite of these constraints, we quantified the degree of similarity between each ensemble of surrogate networks and the cortical network, and we also examined the similarity within each surrogate ensemble.

To examine topological similarity, we calculated the mean binary and weighted similarity quotients, QS^b^ and QS^w^ (Eqs. 1 and 2) of the networks in the three surrogate sets to the cortical network. For random surrogates, QS^b^(C, S_rnd_) = 0.054±0.002 and QS^w^(C, S_rnd_) = 0.047±0.002, indicating that their connections are almost entirely different from those of the cortical network. For spatial surrogates, we obtained intermediate similarity quotient values QS^b^(C, S_S_) = 0.494±0.002 and QS^w^(C, S_S_) = 0.483±0.002, and for reduced surrogates higher similarity quotients QS^b^(C, S_R_) = 0.670±0.001 and QS^w^(C, S_R_) = 0.700±0.001. These results confirm that, as expected, conserving and, even more significantly, further decreasing the already short connection lengths of the cortical connectivity network limits the achievable topological randomisation of the spatial and reduced surrogate networks.

The similarity quotient values described above exhibit only very small deviations around their respective means. This could reflect the combined consequence of a sufficiently extended connection shuffling process together with the relatively large size of the networks, following the law of large numbers. But it could also indicate an undesirably low diversity in the generated surrogate sets, i.e., each set might be composed of highly similar networks. To test for this possibility we calculated the similarity quotient between every pair of surrogate networks in each of the surrogate sets. The resulting mean intra-group values and their standard deviations are QS^b^(S_rnd_, S_rnd_) = 0.053±0.001 and QS^w^(S_rnd_, S_rnd_) = 0.045±0.001 for random surrogates; QS^b^(S_S_, S_S_) = 0.474±0.003 and QS^w^(S_S_, S_S_) = 0.483±0.002 for spatial surrogates, and QS^b^(S_R_, S_R_) = 0.873±0.002 and QS^w^(S_R_, S_R_) = 0.861±0.002 for reduced surrogates. Together, these results indicate that topological differences among surrogate ensembles, although decreasing with stricter spatial constraints, are nevertheless significantly nonzero.

Interestingly, the low intra-group variance of the similarity values within every surrogate set suggests that in each such set S there is a ‘characteristic similarity’, QS(S,S), between any two members of that set. In addition, the similarity of the cortical network to its surrogate networks is comparable to these characteristic intra-group similarities in the case of random and spatial surrogates (QS(C, S_S_)≈QS(S_S_, S_S_) and QS(C, S_rnd_)≈QS(S_rnd_, S_rnd_)). This suggests that the cortical network is a generic member of the random and spatial surrogate sets in terms of its basic region-to-region connectivity, as measured by QS. This further supports the use of random and spatial surrogates as suitable null-hypothesis networks with respect to the preserved basic properties of the cortical connectivity defined by each surrogate type.

By contrast, reduced surrogates appear to form a separate class of networks that are more similar to each other than to the cortical network (QS(C, S_R_)≪QS(S_R_, S_R_)). This is expected given the restrictive form of spatial constraint applied during their generation (strictly decreasing total connection length in every rewiring step), which is likely to make them collectively drift away from their cortical origin, converging towards the (hypothetical) single, minimal connection length surrogate network.

The QS values illustrate well the highly optimised wiring of the cortical network in terms of connection length. While random surrogate networks share only 5.4% of their connections with other random surrogates and with the cortical network, this ratio increases to 49.4% for spatial surrogates, and each reduced surrogate is only able to substitute about one third of the long-range cortical connections with shorter ones. Furthermore, as shown in Figure 3A–C, these pair-wise overlaps translate into a ‘core’ set of connections collectively shared between the cortical network and its spatial and reduced surrogates. This ‘skeleton connectivity’ is primarily located along the main diagonal of the connectivity matrices, where most of the potential short-distance connections can be placed (due to the spatial ordering of the brain regions in the connectivity matrices, explained in detail in the caption of Figure 3).

We note, however, that Figure 3B–D show the averages of the connectivity matrices of the surrogate network groups and therefore exaggerate the pair-wise overlap of the networks in each group. This is a consequence of the relatively small set of potential short-range connections in cortical space (compared to the number of all possible connections), a number of which are inevitably shared by many reduced and spatial surrogates. For example, to examine the most extreme case of shared connectivity, we can determine the connections that are present in all network instances of each surrogate group. As expected, there are no such collectively shared connections among random surrogates. On the other hand, the highly optimised, and hence self-similar, reduced surrogates collectively share as many as 65.0% of their connections, while the ‘intermediately’ constrained spatial surrogates have only 7.6% of their connections shared among all of them, rendering the latter surrogate group relatively diverse. Furthermore, all shared connections of reduced and spatial surrogates are also present in the cortical network. These findings, in accordance with the ones on QS above, indicate that the cortical network is indeed a generic member of its spatial (and random) surrogates in terms of the basic properties of its connectivity, adding some topological credibility to our surrogate analysis.

### Validation of surrogate networks: Spatial similarity

Having assessed the *topological* similarity of the surrogate ensembles to the cortical network, we now investigate the other relevant aspect of surrogate creation, namely to what degree the *spatial* layout and wiring properties of the cortical network have been changed in the surrogate ensembles. Although topological and spatial similarity are related, they do not specify each other. For example, low *topological* similarity between the cortical network and its surrogates in itself does not exclude that connections of the cortical network may only have been displaced by a short distance, leaving the *spatial* layout of the network largely unaffected by the randomisation procedure.

In order to assess the impact of the randomisation procedure on the spatial layout of the cortical network, we calculated the relative spatial displacement D between the high-resolution cortical network and its surrogate groups (see *Assessing spatial similarity* in *Methods*). We obtained a D(C,S_rnd_) = 4.04±3.43 mean displacement value for random surrogates, indicating that on average a neighbour *l* of each region *r* in the cortical network is replaced by a new neighbour *m* in random surrogates, which is about four times further away from the original cortical neighbour *l* than the length of the original cortical connection (*r*,*l*). In spatial and reduced surrogates, we measured D(C,S_S_) = 0.50±0.62 and D(C,S_R_) = 0.29±0.43, respectively, indicating a necessarily lower mean spatial displacement of the regions' neighbourhoods in the topologically more similar spatially constrained surrogates. However, because a significant number of connections is shared by the cortical network and its surrogates (see *Topological similarity of surrogate networks*) and hence have zero displacement, the high standard deviation in D(C,S_S_) and D(C,S_R_) indicates that those connections that have actually been rewired are displaced to a location that is substantially distant from their original target region in the cortical network. This is indeed what we see if we exclude the overlap of the connectivities and calculate the spatial displacement D^r^ of the replaced connections only: D^r^(C,S_S_) = 0.97±0.57 and D^r^(C,S_R_) = 0.88±0.30, which indicates that the average displacement of rewired connections is almost as large as the length of the original connection.

### Connection lengths

The connection length distribution and total connection length of each region (sum of distances to all neighbours) in the high-resolution cortical network and its surrogates are shown on Figure 3E and F. Consistent with the predominantly local connectivity of the cortical network (mean connection length per region: CL_C_ = 27.625 mm), random rewiring of cortical connections nearly tripled the average connection length (mean ± standard deviation of random surrogate network means: CL_rnd_ = 75.971±0.164 mm). For this reason, that is, due to the natural tendency of random connection swapping to increase the length of originally short cortical connections, the simple condition applied during spatial surrogate generation (i.e., ‘not to exceed the original total connection length of the cortical network’) was sufficient to actually achieve *conservation* of connection lengths (CL_S_ = 27.507±0.120 mm), and resulted in a slightly narrower connection length distribution (standard deviation of connection lengths: cortical network: σ^l^_C_ = 22.146 mm→spatial surrogates: σ^l^_S_ = 18.589 mm) originating from a somewhat shorter tail of the distribution (see Figure 3E).

Wiring length optimisation in *reduced* surrogates of the high-resolution weighted network successfully reduced the mean cortical connection length by 29.6% (CL_R_ = 19.433±0.013), effectively substituting long-range cortico-cortical projections with shorter, local ones. This also led to a much narrower distribution of connection lengths (standard deviation of connection length: cortical network: σ^l^_C_ = 22.146 mm→reduced surrogates: σ^l^_R_ = 7.382 mm). As a result of the above, the total connection lengths of *individual* cortical regions were preserved in spatial surrogates (cortical network – spatial surrogates mean difference: −2.4±5.5%, Wilcoxon rank-sum test for identical distribution: p = 0.898), while reduced and random surrogates had significantly decreased (−24.6±17.0%) and increased (+227.6±114.0%) regional connection lengths, respectively (p<10^−4^ in both cases).

### Comparison with other minimally wired models

Several earlier studies investigated spatially minimally wired surrogates of various neural and brain connectivity networks [51]–[54]. In order to investigate how much excess wiring length cortical connectivity has over its theoretical minimum, as well as to assess how the reduced surrogates compare to ‘bottom-up’ constructed, minimally wired models, we assembled two such models.

For the first, unconstrained minimally wired network model, which we call absolute minimal (AM) network, we took the 998 cortical regions without their connections and simply placed links between the 17865 spatially closest region-pairs. This created a network with minimal total wiring length given the spatial arrangement of the cortical regions and the total number of connections in the cortical connectivity. The resulting AM network is composed of a single component (no disconnected regions or groups of regions). The sum of its connection lengths is 62.9% of that of the cortical network, which, importantly, is only 10.6±0.1% less than the total connection lengths of the reduced surrogate networks. Importantly, the degree distribution of the original cortical network has been completely lost in the AM network (mean relative deviation of regional degrees between cortical network and the AM network: 52.5±130.7%). This means that the reduced surrogates were able to achieve highly optimised wiring lengths while fully preserving the cortical network's degree distribution, thus providing a powerful topological baseline to assess the significance of the cortex's high level network properties. Both the cortical network and its reduced surrogates share a large number of their connections with the AM network (binary similarity quotient: QS^b^(C,AM) = 0.621, QS^b^(S_R_,AM) = 0.760±0.0009), showing once again the remarkably conservative wiring of the cortex: 62.1% of the cortical connections are among the theoretically shortest possible links in the cortical network.

We devised a second ‘bottom-up’ constructed minimally wired connectivity model with the additional constraint of approximating the degree distribution of the cortical network. We construct this network, which we call the degree preserving minimal (DPM) network, in the following way. As with the AM model we start with the 998 cortical regions without any connections, and, by going through the list of potential connections (region-pairs) ordered from shortest to longest, we add each connection to the DPM network only if the current degrees of both corresponding regions in the DPM network are less than their original degrees in the cortical network. By this simple strategy we are able to create a network with 17799 connections (66 connections [0.4%] less than the cortical network) that closely approximates the degree distribution of the cortical connectivity (mean percentage deviation in regional degrees between cortical network and the DPM network: 0.2±1.8%). Due to the similarity in degrees, the DPM network shares an even larger number of connections with both the cortical network and the reduced surrogates than the AM network (binary similarity quotient: QS^b^(C,DPM) = 0.653, QS^b^(S_R_,DPM) = 0.855±0.002). The sum of connection lengths in the DPM network is 72.1% of that of the cortical network, which is on average 2.4±0.1% *more* than those of the reduced surrogates, despite the fact that the DPM network has slightly less connections than the reduced surrogates. This demonstrates that simple ‘bottom-up’ algorithms are not guaranteed to be more successful in constructing minimally wired (surrogate) networks than the rewiring methods used in the current study.

We conclude that the spatial surrogates effectively preserved the wiring length properties of the cortex, both globally and at the level of individual regions, and that the reduced surrogates significantly decreased wiring length by substituting long-range connections with shorter ones. These properties render spatial and reduced surrogates suitable for representing a wiring-length-matching and wiring-length-optimised null-hypothesis network set of the cortical connectivity, respectively. The results so far demonstrate that, as opposed to the highly unrestricted nature of random surrogates, the presence of strict wiring constraints necessarily limits the form of potential connectivities of the cortex at the basic level of region-region connections, as shown by elevated similarity between cortical network and its spatial and reduced surrogates as compared to random surrogates.

In the remainder of the paper, we go beyond these basic properties, to examine which other, network-level properties of the cortical connectivity these wiring constraints preserve. We measure the degree of expression of these properties by a series of complex network metrics, in each case applying the interpretations detailed in the *Introduction* (see also Table 1).

### Integration, segregation and small-worldness

The need for the simultaneous presence of functional integration and segregation imposes conflicting constraints on network architecture [55], reflected in properties collectively known as ‘small-world’ characteristics. Small-world properties have been found in many real-world complex networks [23], including various brain networks [10], [56]–[58].

**Figure 4:**
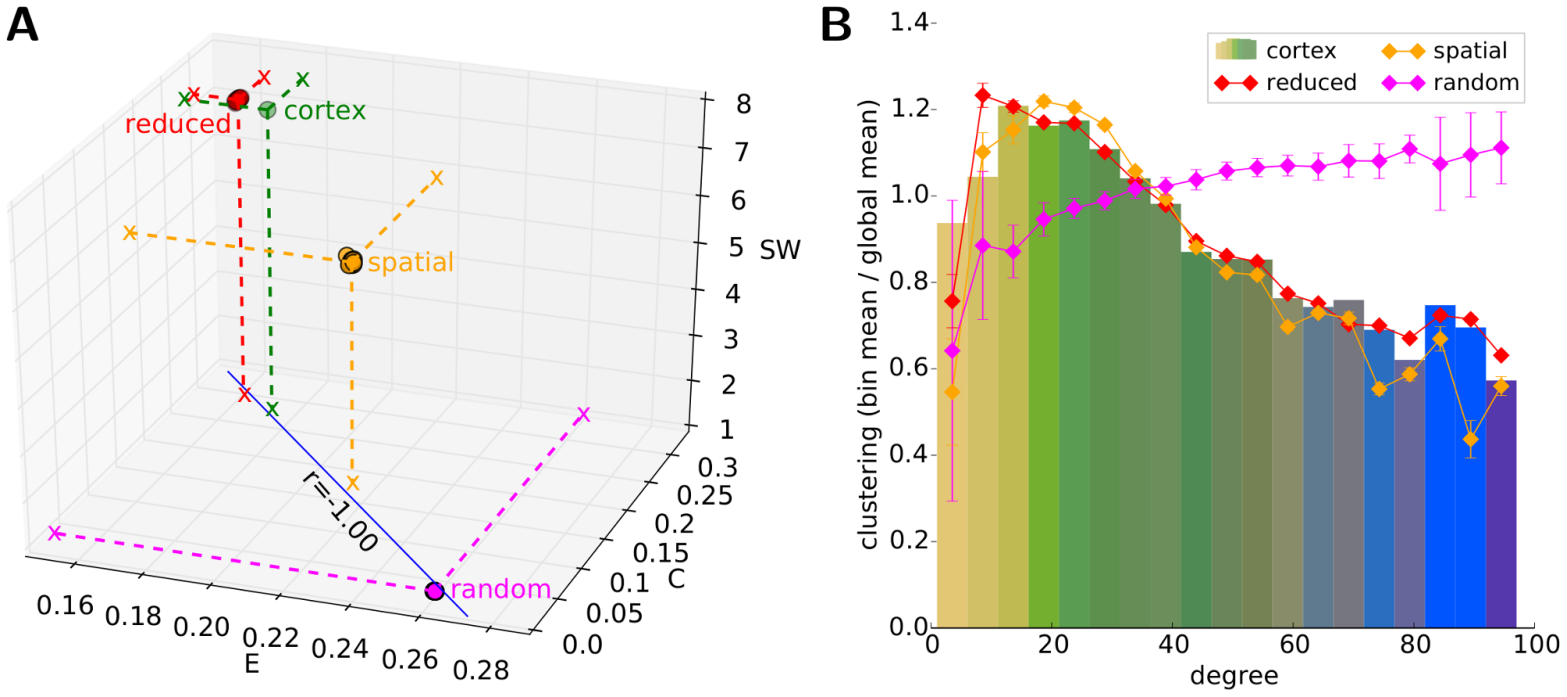
Integration, segregation, small-worldness and hierarchical organisation. (A) Relation between clustering coefficient (C), global efficiency (E) and small-world (SW) index in high-resolution cortical connectivity and its surrogate networks. Each coloured sphere represents a single network (the cortical or a surrogate network), with dashed lines guiding the eye to their projections on side panes. Random surrogates lay on the E–C pane with small-world index SW_rnd_ = 1 by definition. Note the remarkably similar values of the individual surrogate networks within each surrogate group for all three measures, indicated by the closeness of their corresponding spheres. Also note the strong negative correlation between clustering coefficient and global efficiency across all investigated networks (blue line). *r*: Pearson correlation coefficient. (B) Relation of nodal degree and clustering coefficient. Regions are binned by degree (*x* axis), and plotted against the average clustering coefficient of the bin normalised by the global average clustering coefficient of the network (to bring all networks to the same scale). Bars correspond to cortical results and are colour-coded to the mean of the colours of the regions they contain (see sector names in Figure 2A). Diamonds and whiskers represent surrogate mean values and standard deviations (see legend). Bar colours indicate the abundance of temporal regions at low degrees, frontal regions at medium degrees, and parietal, limbic and occipital regions at high degrees. This structural differentiation across degree range holds for all the surrogate networks due to the identical degrees of their regions. Note the negative correlation between clustering and degree in the cortical network and the reduced and spatial surrogates, suggesting their hierarchical organisation, as opposed to the positive correlation in random surrogates.

We measured the global integration and segregation potential of the cortical network compared to its surrogates using the quantities efficiency E and clustering coefficient C (see *Methods*). As shown in Figure 4A, the cortical network is more similar to its reduced surrogates than to its other two surrogate sets (high-resolution weighted cortical network: E_C_ = 0.174, C_C_ = 0.271, reduced: E_R_ = 0.162±0.001, C_R_ = 0.289±0.002, spatial: E_S_ = 0.214±0.001, C_S_ = 0.169±0.002, random: E_rnd_ = 0.260±0.001, C_rnd_ = 0.024±0.001). Considering that the total connection length of each region in the cortical network is the same as in its spatial surrogates, and that long-range connections are largely absent in reduced surrogates, the efficiency results indicate that the long-range cortico-cortical connections are distributed in a topologically sub-optimal way for enhancing tight functional integration (efficiency) in the cortical network. Furthermore, the clustering coefficient indices demonstrate a prevalence of topologically segregated neighbourhoods of groups of regions, beyond what would be expected from the wiring constraints of its individual regions (C_C_ is significantly larger than C_S_ and much closer to C_R_ than to C_S_). Therefore, not only when comparing against the necessarily more highly integrated and less segregated random surrogates, but also when taking into account the total length of the connections of each cortical region in the spatial surrogates, the cortical network appears to strongly favour topological segregation over integration (efficiency).

In order to assess the effect of wiring constraints on its small-world attributes, we calculated the small-world index SW of the cortical network and its spatial and reduced surrogates (see *Methods*), using random surrogates as reference networks (random surrogates hence have SW_rnd_ = 1 by definition). First we note that all three investigated network types (cortical network, spatial and reduced surrogates) satisfy the basic small-worldness condition [37], that is, their clustering coefficient is larger than those of its random surrogates (C≫C_rnd_) while their efficiencies (average shortest path lengths), while being lower (higher), are still comparable to those of their random surrogates (E≈E_rnd_). In case of the cortical network, this results in the relatively high small-world index SW_C_ = 7.478 (see Figure 4A), indicating a well-expressed small-world organisation of the cortex. In comparison, we obtain on average SW_S_ = 5.746±0.031 for spatial surrogates, and SW_R_ = 7.419±0.031 for reduced surrogates, both much closer to SW_C_ than the random surrogates (recall SW_rnd_ = 1), indicating that the small-world architecture of the cortex can be attributed to a great extent to its wiring constraints. However, considering that SW_C_ is significantly higher than SW_S_, the cortical network appears to exhibit the small-world property beyond what would be implied by its local connectivity alone. Furthermore, this excess level of cortical small-world organisation does not necessitate any particular arrangement, or even the presence, of the long-range cortical connections, as indicated by SW_R_ not being significantly different from SW_C_. Therefore, the highly segregated connectivity of the cortical network, also found in reduced surrogates, but not in spatial surrogates (see above), appear to contribute more to the small-world organisation of the cortex than the mere existence or particular arrangement of cortical long-range connections.

### Hierarchy

In their seminal work, Ravasz and Barabási [38] detected well-expressed hierarchical structure in all investigated non-spatial (non-geographical), real-world networks, but not in spatial examples (e.g. the power grid network and the Internet). They reasoned that the high cost of establishing physically long connections substantially limits the type of topology spatial networks can exhibit, potentially excluding strongly hierarchical forms. However, in a study of a 104-region structural network of the human cortex Bassett et al. [10] did find hierarchical properties in the brain among multimodal cortical regions, but not within unimodal and transmodal regions.

Following Ravasz and Barabási [38] (see *Methods*), we calculated the average clustering coefficients of groups of cortical regions with similar degrees, relative to the global clustering coefficient of the cortical network (see Figure 4B). We observe that the cortical network exhibits a steep decline in its mean clustering – degree relation, indicating that the cortex exhibits the type of hierarchical organisation illustrated in Figure 1B. This finding supports the general notion of a hierarchically organised brain [59], which is quite remarkable given the tendency of spatially embedded, physical networks not to develop hierarchical features due to the basic spatial (geographical) constraints acting on them [38]. Furthermore, there are highly similar tendencies for spatial and reduced surrogates, but not for random surrogates, in which clustering actually increases with region degree. The remarkably high consistency of the clustering – degree relationship across the cortical network and its spatial and reduced (but not random) surrogates indicates that the individual wiring lengths and positioning of high degree regions in the cortex by itself entails a global hierarchical organisation.

### Modularity

Many real world networks have a characteristic topology that allows them to be separated into relatively densely intra-connected and weakly inter-connected subgroups [16], [60]. These subgroups are usually referred to as the modules (or clusters, communities) of the network. One possible functional advantage of modularity is reduced systemic risk during development and evolution [61], [62]. Another is that modular architectures are related to potentially useful dynamical properties such as high dynamical complexity [21] and metastability [63], as well as limited sustained network activity [64].

Recent studies have reported a highly modular architecture of the human brain in its structural [12], [13], [65] as well as in its resting state functional connectivity (rsFC) [66]–[68]. Furthermore, studying the effect of ageing on the brain's modular structure, Meunier et al. [69] found marked differences in the composition and putative topological roles between the modules in the rsFC of younger and older human subjects. These results suggest that modular ‘decomposability’ is a prominent feature of the brain, which is continuously shaped during its development, maturing and ageing. In line with these results, recent theories regard the brain's modular structure as the main facilitator of regional specialisation and segregated functional processing [18].

**Figure 5:**
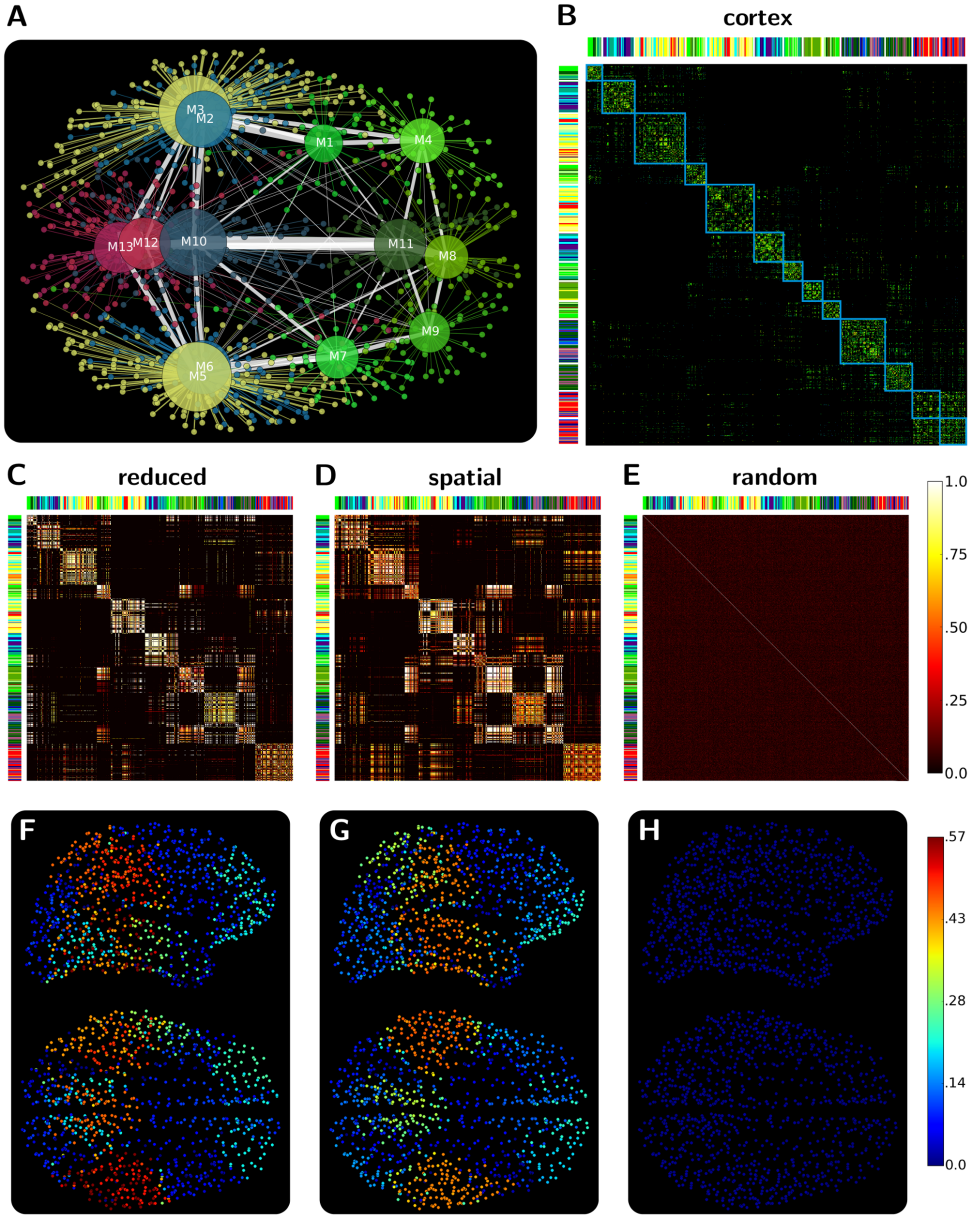
Module organisation. (A) Illustration of the identified cortical modules on a horizontal projection. Modules, represented by large circles, are drawn at the mean position of the regions they contain, with a radius proportional to their size (number of their regions), and are coloured by the average ‘anatomical structure colour’ (see section labels in Figure 2A) of their regions. Regions (smallest circles) are connected to their modules, and drawn with the colour of their modules. The widths of the inter-module connections (white lines) are proportional to the sum of connection strengths between regions in each of the two modules to regions in the other. (B) high-resolution cortical connectivity matrix ordered according to recovered modules (blue sub-squares along main diagonal, from M1 to M13 from top left to bottom right). Left and top colour stripes show colour-code of the corresponding cortical region's greater anatomical structure (see section labels in Figure 2A). For colour-code of matrix elements see colour-bar in Figure 3. (C–E) Module correspondence matrices of the three surrogate groups. Each element of the matrices denotes the normalized frequency (from black through red to white, see colour-bar on right side) of the two regions to be placed into the same module in the module partitions found in the corresponding surrogate network group. (F–H) Consistency of cortical module partition in surrogate groups. Consistency of module classification of each region is measured by its mean scaled inclusivity in each surrogate group using the obtained cortical modules as reference partition (see *Methods*). Colour-coded scaled inclusivity values of regions are shown on coronal (top) and horizontal (bottom) projections (see colour-bar on right side).

We investigated the modular structure of the cortical network and its surrogates by utilising Newman's module detection algorithm [41] (see *Methods*). In order to assess the strength of modular organization, that is, the magnitude of the Q modularity index, we use the modularity of the random surrogates as a baseline value (representing the modularity index of a non-modular network with size and connection density matching that of the cortical network). These random surrogates, as expected due to their quasi-zero segregation, express almost no modularity (mean modularity index: Q_rnd_ = 0.087±0.003, number of modules: N_rnd_ = 23.25±1.95). In contrast, the cortical network has a strongly modular architecture (Q_C_ = 0.558) composed of N_C_ = 13, spatially compact and hemispherically symmetric modules (Figure 5A). Interestingly, reduced surrogates, in spite of their lack in long-range (thus mostly inter-module) connections, do not exhibit a significantly higher modularity index (Q_R_ = 0.567±0.015, N_R_ = 15.55±0.87, one-tail t-test assuming normal distribution: p = 0.274), but spatial surrogates do possess a significantly lowered level of modularity (Q_S_ = 0.477±0.020, N_S_ = 11.55±0.87, p<10^−4^) than the cortical network. These results show that while the physically constrained length of cortico-cortical white-matter connections are a fundamental factor in shaping the high strength (Q_C_) and granularity (N_C_) of the global modular architecture of the cortex, the cortical network nevertheless has a stronger modular organisation than these wiring constraints by themselves would suggest, indicating the functional relevance of the cortex's modular structure.

The strength of the modular organisation of the cortical network can be illustrated by its inter- versus intra-modular connection distributions (Figure 5B). The N_C_ = 13 identified modules contain 63.2% (n = 11294) of the total number of projections internally, meaning that only 36.8% (n = 6571) of the connections cross module boundaries. This results in an average 25.6% intra-module and 1.4% inter-module connection density, indicating that while more than every fourth intra-module region-pair is linked, this ratio falls to 1∶70 for region-pairs from different modules. For comparison, the global average connection density of the entire network is 3.6%.

The cortical connectivity matrix ordered by the recovered module partitioning is shown in Figure 5B. To compare this partitioning with an ‘average’ partitioning for each surrogate group, we calculated the frequency with which every region-pair (n_i_, n_j_) can be classified into a single module (m(n_i_) = m(n_j_)) in each of the three surrogate network groups. The resulting matrices are shown in Figure 5C–E. The high concentration of frequent co-partitioning of region groups along the main diagonal of the matrices is apparent in the case of reduced and spatial surrogates, indicating that the corresponding cortical modules are reasonably preserved across these surrogate networks. Furthermore, there is also a tendency for the formation of off-diagonal blocks in Figure 5C and 5D which suggests that parts of some of the cortical modules are frequently merged into single surrogate modules, and therefore they are at least partially preserved in reduced and spatial surrogates.

Motivated by these findings, we quantitatively tested the robustness of the modular partitioning of the cortical network against the rewiring applied to its surrogate groups by assessing the consistency of the cortical partition in the surrogate groups. To do this, we used the obtained cortical modules as a reference partition and measured the scaled inclusivity index SI of each cortical region in the surrogate groups (see *Methods*). Among the three surrogate sets, reduced surrogates exhibited the highest mean SI index, indicating the highest overall conservation of cortical modules in reduced networks, although with high variations across the individual cortical regions (mean ± std: reduced surrogates: SI_R_^C^ = 0.235±0.182, spatial: SI_S_^C^ = 0.202±0.145, random: SI_rnd_^C^ = 0.007±0.002).

The SI values for the individual cortical regions, and for each surrogate group, are illustrated in Figure 5F–H. We found elevated robustness of the cortical modules in both reduced and spatial surrogates at specific cortical sites, including the entire pre-central and post-central cortices (composing cortical modules M2 and M6 on Figure 5A), large areas of the temporal lobe (M3 and M5) and some frontal (M4 and M9), and superio-parietal and limbic areas (M10). The high SI of these specific areas indicates that their modular structure exhibits greater robustness against spatially constrained rewiring, as opposed to the low SI of, and thus higher variance in, the module formations in other parts of the cortical network.

### Core formation

The results so far, regarding the small-world, hierarchical and modular architecture of the cortex, suggest the existence of specific cortical areas that are topologically centrally positioned in the modular structure of the cortical network. This ‘core formation hypothesis’ has been the topic of several studies recently (see below), and we next test its significance against the wiring constraints of the cortex by again analysing the surrogate ensembles.

Intuitively, the core of a network, illustrated in Figure 1C, is a set of ‘elite’ nodes that are topologically centrally positioned, forming a highly intra- and inter-connected global centre [28]. The existence of a single, but strong core formation in the topology of a network typically suggests that the network exhibits a pronounced global core-periphery structure [70]–[72] and indicates the presence of centralisation in the network's dynamics and functional operation, which is fundamentally different from that of a homogeneous network architecture composed of distributed, identically segregated units (e.g., Figure 1A).

Prior studies have identified and investigated a core structure in various brain networks, including the rich-club structure of the cat thalamo-cortical complex [17], , the k-core of the macaque brain [75], the s-core of the human cortex [12], and the rich-club of the entire human brain [13], [25]. We here compare s-core and rich-club properties of the cortical network and also assess the extent of their dependence on, and emergence given, different wiring constraints using the three surrogate types.

### S-core significance

**Figure 6:**
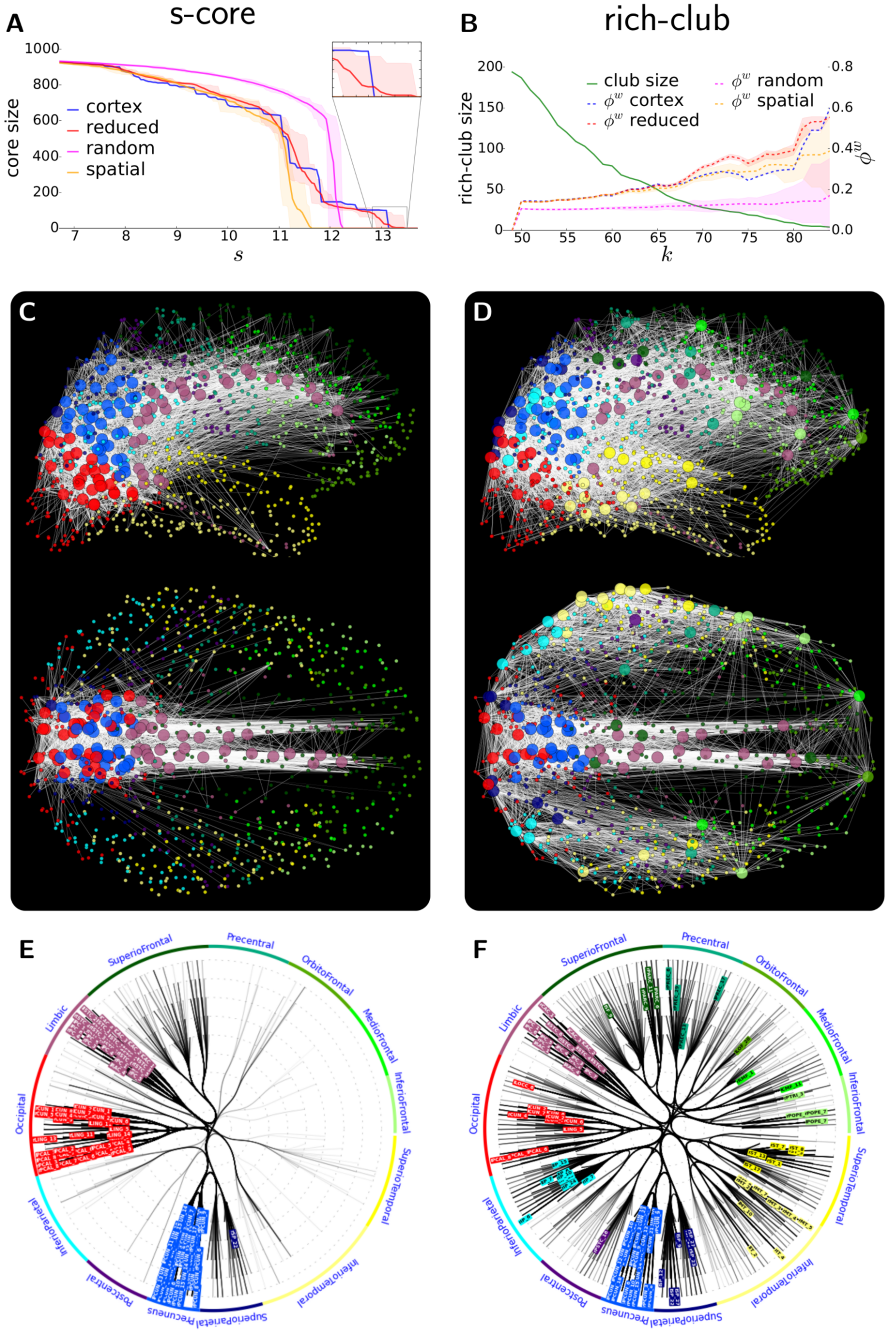
S-core and rich-club analysis. (A) S-core size as the function of core strength threshold *s* in the cortical network and its surrogates. (B) Weighted k-density φ^w^(k) (dashed lines) and rich-club size (solid line) as the function of degree threshold *k* in the cortical network and its surrogates. The rich-club size (green) is the same for all networks due to their identical degree distribution. Colour-filled intervals indicate the full range of values observed in surrogates. (C–F): cortical regions of the final s-core (n = 100 regions at s = 13.095) and the equivalent size rich-club (n = 100 regions at k = 57) and their connections are visualized on coronal (top of C and D) and horizontal (bottom of C and D) projections, and on an abstract hierarchical radial layout (E and F).

S-core analysis assesses the extent to which a network exhibits a densely intra-connected inner core, by measuring the size of, and overall connection strength within, the most strongly intra-connected group of nodes. We identify the s-core of the cortical network through a ‘peeling’ procedure that iteratively removes less connected regions from a candidate s-core (see *Methods*). Examining the evolution of the s-core decomposition of the high-resolution cortical network and those of its surrogates (Figure 6A) during the peeling procedure, we can identify two characteristic phases. A longer, rather stable early phase of ‘crust peeling’ transitions into an unstable phase for s>11, in which the s-cores of both random and spatial surrogates diminish rapidly and then abruptly vanish. The cortical network, on the other hand, closely follows the trend of its reduced surrogates and continues to sustain a substantial s-core of n = 100 regions (10.0%) for much longer. This s-core eventually collapses at a significantly higher strength threshold (s_C_ = 13.095) than its counterparts in the random (s_rnd_ = 12.055±0.078) or spatial surrogates (s_S_ = 11.433±0.124), within the range of the s-cores of reduced surrogates (s_R_ = 13.027±0.143), but with a somewhat larger size (s-core size of cortical network S^C^ = 100, reduced surrogates: S^R^ = 74.500±17.119, see Figure 6A inset). Considering that the connectivity of reduced surrogates is spatially more concentrated than that of the cortex, which is a property that favours the formation of a strong s-core, the above finding suggests that cortical connectivity may be optimised towards the formation of a global s-core, which is much stronger and larger than its connection length constraints alone would suggest.

### Rich-club significance

An alternative measure of core formation in a network is the assessment of its rich-club index [47], [48]. The weighted variant of a rich-club index, φ^w^(k), measures the tendency of high degree nodes to be both densely and strongly inter-connected (see *Methods*). Examining the evolution of φ^w^(k) with increasing *k* in the cortical network and in its surrogates (Figure 6B), the cortical network demonstrates a rich-club of significant strength (weighted k-density at n = 100 regions: φ^w^_C_(100) = 0.164) compared to its random surrogates (φ^w^_rnd_(100) = 0.106±0.002). However, the cortical network does not possess a significantly stronger rich-club structure than its reduced surrogates (φ^w^_R_(100) = 0.164±0.001, one-sample t-test: p = 0.23) or its spatial surrogates (φ^w^_S_(100) = 0.163±0.003, one-sample t-test: p = 0.34).

Previous studies [13], [17], [25] used only random surrogates as null-hypothesis baselines for assessing the rich-club property of brain networks, a comparison in which the cortical networks we study here also express a highly developed rich-club (Figure 6B, compare blue and magenta lines). However, we show here that this property is equally, or even more, expressed in spatial and reduced surrogates. Closer inspection reveals that the relatively low variance in the spatial locations of highly connected regions (Figure 6D and F), in combination with the highly clustered, local connectivity of the cortex, naturally results in a tendency for strong rich-club formation.

The wiring-constraint-dependent rich-club formation tendency of the cortex is further supported by the assortativity coefficients *r* of the network and its surrogates (see *Methods*). We found significantly positive assortativity coefficients for the high-resolution cortical network (*r*_C_ = 0.288) and its spatial (*r*_S_ = 0.283±0.004) and reduced surrogates (*r_R_* = 0.326±0.002), indicating their tendency to connect nodes of similar degree, whereas almost no degree assortativity is found in random surrogates (*r*_rnd_ = 0.051±0.006). This preferentially mutual connectedness of high degree regions suggests that the rich-club patterning of the cortical network naturally arises from the physical location of cortical hubs and the cortical wiring constraints.

The s-core and rich-club regions selected by the two methods (Figure 6C–F), are largely consistent with earlier findings [12], [13]. Furthermore, the s-core (n = 100 regions in final, non-empty core) and rich-club regions (n = 100 highest degree regions) exhibit a considerable, exactly 50% (n = 50 regions) overlap in the cortical network. There are, however, marked differences in the anatomical composition and spatial dispersion of the two structures. The s-core of the cortical network encapsulates the caudal part of the cortical midline, formed by the precuneus, the cingulate cortex and the superior part of the occipital lobe (cuneus, lingual gyrus and pericalcarine cortex). This centralisation is also present, though much less pronounced, in the cortical network rich-club, since about one third of it extends to the lateral and frontal parts of the cortex. The spread of arborisation of the two cores also exhibits this difference (see Figure 6C–F): while the more numerous (n = 5662 [31.7%] connections) and rather externally projected connections (20.6% internal connection density) of the rich-club establish direct connectivity with almost the whole remainder of the cortex (n = 795 [88.5%] regions), the s-core possesses a smaller (n = 3921 [21.9%] connections), as well as more internally projected connection set (37.7% internal connection density), which connects it directly with only one third (n = 294 [32.7%] regions) of the rest of the network. These differences, originating from the definitions of the s-core and rich-club structures, demonstrate the more distributed nature of the cortex's rich-club, as opposed to the rather encapsulated, but spatially and topologically central position of the s-core.

### Results on low-resolution and binary connectivity types

Along with the analysis on the high-resolution weighted version of the cortical connectivity dataset presented above, we also performed our surrogate analysis on four ‘subsets’ of the full dataset, namely: on the binarized (unweighted) version of the high-resolution cortical network, on the weighted and the binarized versions of a lower resolution (down-sampled) cortical network (see *Methods*), and on a single hemisphere extracted from the high-resolution weighted cortical network (discussed in detail in the following section).

**Figure 7:**
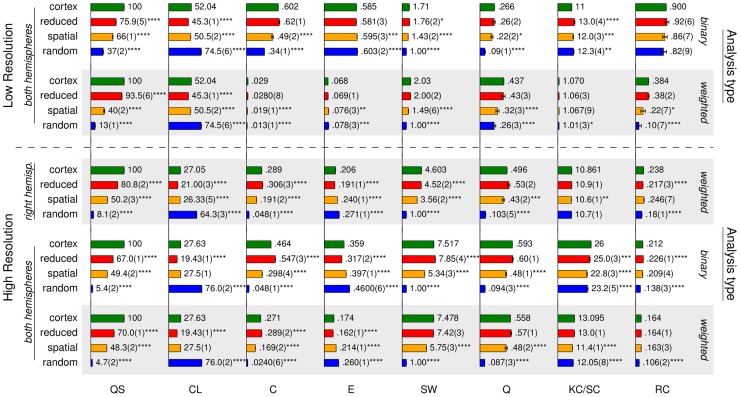
Summary of analysis. Results are given for all weighted and binary (unweighted) analyses performed on both high- and low- resolution cortical connectivity, as well as on a single high-resolution hemisphere. In main text we focus primarily on the weighted analysis of high-resolution network (bottom gray section). Bar heights indicate cortical values and surrogate group means, whiskers show standard deviations across surrogate networks of the same type (negligible for most measures). Number after each bar shows corresponding mean value up to the first digit with non-zero standard deviation value, which is given in following parentheses. QS: Sørensen similarity quotient, C: clustering coefficient, CL: average connection length (Euclidean distance between connected region-pairs in mm), E: global efficiency, SW: small-world index, Q: modularity index, KC/SC: final strength of k-core (binary analysis) and s-core (weighted analysis), RC: rich-club strength (binary or weighted k-density) of 100 (high-resolution) and 10 (low-resolution) highest degree regions (hubs). Stars denote that the cortical network is statistically significantly different from its surrogate ensemble (one sample t-tests): *: p<0.05, **: p<0.01, ***: p<0.001, ****: p<0.0001.

Similarly to the analysis of the high-resolution weighted cortical network, we first tested the surrogates of the three cortical networks considered here with the topological similarity measure QS and the measure of mean connection lengths per region, CL. Our surrogate test results on the three cortical networks showed the same pattern that we described for the weighted high-resolution cortical network (see Figure 7), albeit with an overall lower level of randomisation (higher topological similarity) for the low-resolution networks, due to the higher connection density of these networks (high-resolution: 3.6% connection density, low-resolution: 26.8%), as well as a slightly (but significantly) reduced connection length in low-resolution spatial surrogate networks, likely due to the limitations of re-wiring algorithms on smaller networks.

Next we assessed the global integration and segregation potential of these cortical networks by calculating their clustering coefficient C and efficiency E, respectively. In accordance with the high-resolution weighted results, we found the same pattern of higher similarity of each cortical network to its reduced than to its spatial surrogates consistently across all analysed cortical networks, to the extent that for low-resolution networks there is no significant difference between them (see Figure 7). Therefore, as with the high-resolution weighted cortical network, these networks also demonstrate a small-world index more similar to their reduced than spatial surrogates (see Figure 7). Surrogate analysis of the modularity strength Q of these cortical networks also yield highly consistent results with those of the high-resolution weighted cortical network (see Figure 7). Taken together, these findings are consistent with our results on the high-resolution weighted cortical network; they indicate that the functional segregation potential and the small-world and modular organisation of the connectome, even when observed on lower resolutions, are significantly stronger than its wiring constraints alone can account for.

We next evaluated the core formation tendencies of the three cortical networks. Results on the k-core (unweighted s-core) of the high-resolution *binary* connectivity are in agreement with the high-resolution weighted network results discussed above. On low network resolution, however, we observe different characteristics (see Figure 7). Specifically, the significantly strong k-core and s-core structures of the binary and weighted high-resolution cortical networks seem to weaken to a weighted low-resolution cortical s-core of comparable strength to its wiring-constrained surrogate ensembles, and further diminish to a binary low-resolution k-core with a strength significantly weaker than any of the surrogates. When investigating the binary low-resolution cortical k-core more closely, we discovered that it contains as much as 80.3% (53 regions) of the entire network, and any subsequent peeling step (see *Methods*) destroys the whole structure. This is in stark contrast to the k-core of the low-resolution spatial surrogates, which are on average composed of only 52.3% of the network, or with the k-core (s-core) of the binary (weighted) high-resolution cortical network, which contains only 11.2% (10.0%) of the 998 regions. The difference between network resolutions may largely be attributable to the high degree of spatial concentration of the high-resolution s-core (and k-core) regions (Figure 6C and E). This concentration results in the collapse of large parts of the high-resolution core structure into single low-resolution regions of the cortex (specifically into the precuneus, the cingulate cortex and superior areas of the occipital lobe), the extremely dense internal connectivity of which is not accounted for during low-resolution analysis. Consequently, even the weighted, but especially the binary, cortical network, as observed on lower resolution, appear to exhibit a more distributed, homogeneous connectivity, with highly inhomogeneous intra-region connection densities, that are only accounted for at the higher resolution analysis. More generally, these findings underline the importance of multi-resolution analysis in cortical connectivity research in order to obtain a more complete and accurate picture on the inherently multi-level organisation of the connectome.

In conclusion, our surrogate analysis results extend those of Hagmann et al [12] by showing that the core structure of the high-resolution cortical network is both topologically and spatially significant, as measured by both k-core and s-core analysis. Furthermore, our findings on the low-resolution connectivity also indicate that this result depends on high-resolution analysis because the cortical connectivity becomes increasingly sparse and centralised at higher resolutions.

We next evaluated the tendency of the three additional cortical networks for the formation of the other putative ‘core’ structure, the rich-club. In line with the results on the weighted high-resolution connectivity, we obtained cortical network rich-clubs in the low- and high-resolution *binary* connectivities with strengths comparable to those of their spatial surrogates, and even somewhat weaker than those of their reduced surrogates, assessed by the traditional (unweighted) rich-club measure (see Figure 7).

In contrast to these results, we found a rich-club in the weighted low-resolution cortical connectivity that is statistically stronger than those of its spatial surrogates (one-sample t-test: p = 0.02, see Figure 7). Originating from its agglomerative construction from the high-resolution cortical network (see *Methods*), this finding may reflect the highly non-uniform (exponential-like) connection weight distribution of the weighted low-resolution cortical network. In essence, the surrogate rewiring process in the random and spatial surrogates of this cortical network, but not in its reduced surrogates, was effective in relocating the few very short-range, but extremely strong cortical connections to random positions in the network, resulting in a highly variable, but on average lowered weighted rich-club strength in these random and spatial surrogates (Figure 7, second row). (We note that by the nature of their definition, the rest of the weighted metrics investigated in this study, including the s-core structure, are largely immune to this kind of variation in the specific location of these few, extreme strength connections in the low-resolution weighted cortical network.) Nevertheless, the results indicate that the low-resolution weighted cortical network, in agreement with the other three connectivity types, demonstrates a significantly strong rich-club structure, by comparison with traditional random surrogates (one-sample t-test, p<10^−4^). Contrary to the other three connectivity types, however, the strength of the rich-club in the low-resolution weighted connectivity does not seem to be fully attributable to the spatial constraints of the cortex, as indicated by spatial surrogate comparison.

### Single hemisphere analysis

An analysis approximating fibre length by the Euclidean distance of the connected regions (see *Methods*) may disproportionately underestimate the length of the longer curved inter-hemispheric fibres, particularly those connecting homotopic regions around the cortical midline [8]. This, in turn, may result in an increase in the number of inter-hemispheric connections with underestimated lengths in the wiring constrained surrogate networks. Indeed, evaluating the proportion of intra- and inter-hemispheric connections in the cortical network and in the surrogate networks shows that while only 11.5% of the high-resolution cortical connections run between the hemispheres, this ratio increases to 13.2% for reduced, 18.1% for spatial and 50.2% for random surrogates. Some, although certainly not all, of these (surrogate) inter-hemispheric connections are likely to cause a corresponding underestimation in the connection length of reduced and spatial surrogates compared to that of the cortical network. This concern, however, is greatly eased by noting that the regions of the cortex along its midline are already highly intra-connected (see Figure 6), leaving only few potential places where such new connections can be formed. Indeed, calculating the mean (Euclidean) distance between inter-hemispherically connected region pairs D^IH^ on high network resolution, we found an increase, rather than a decrease, in the D^IH^ of spatial surrogates compared to that of the cortical network (D^IH^_ctx_ = 26.2 mm, D^IH^_S_ = 38.8 mm). In comparison, we found, as expected, that the mean distance between the inter-hemispherically connected region pairs is somewhat lowered in reduced surrogates (D^IH^_R_ = 22.2 mm) and greatly increased in random surrogates (D^IH^_rnd_ = 86.2 mm). These results indicate that the newly created inter-hemispheric connections in spatial surrogates are predominantly between relatively distant regions, therefore suffer less from the disproportionate underestimation of connection length, as approximated by Euclidean distance, between homotopic regions along the cortical midline.

Nevertheless, in order to test our results against potential artefacts originating from the different degree of inter-hemispheric connectedness in the cortex and its surrogates, we repeated the analyses using a single cortical hemisphere. Specifically, we extracted the right hemisphere of the weighted high-resolution dataset, generated n = 20 surrogate networks for each of the three surrogate network types using the same method as before, and measured the complex network metrics assessed in the paper.

The results of single hemispheric analysis (Figure 7, third row) are largely in agreement with the previous bi-hemispheric analysis. The main differences are that the (hemi)-cortical network has an increased small-world index compared to reduced surrogates, and its rich-club is slightly but not significantly weaker than those of spatial surrogates (one-sample t-test: p = 0.1), and stronger than those of reduced surrogates. We note that if there was a significant bias in the full cortex surrogate networks to form an excess number of inter-hemispheric connections between homotopic midline regions, we would expect single hemisphere surrogate analysis to detect a consistent *increase*, rather than *decrease*, in the strength of s-core and rich-club structures, given the highly central positioning of these structures along the cortical midline in the full cortical network (see Figure 6). Due the fact that we observe such an increase in only one out of the four possible cases (the rich-club of reduced surrogates), we conclude that the single-hemisphere analysis validates the Euclidean approximation on fibre lengths for our surrogate analysis, and our main conclusions on the bi-hemispheric cortical network appear to largely apply to the uni-hemispheric cortical connectivity as well.

## Discussion

Standard models of complex network science in conjunction with the fundamentals of neuroscience shape the techniques we use for the analysis of brain networks. For example, the abstract concept of small-worldness has traditionally been defined in relation to random and lattice networks [23]. Thus, the diffuse nervous systems of coelenterates (such as *Cnidaria*) have long been recognised to exhibit a characteristically regular, lattice-like pattern [76]. These and other findings have contributed to the wide application of random and lattice surrogate techniques in brain network analysis. In this paper we have investigated how the use of more constrained null-hypothesis models, incorporating not only basic topological but also spatial properties of the human connectome, will help us better understand the structural organisation and functional operation of the inherently spatially and economically constrained brain.

We analysed a dataset representing the large-scale anatomical connectivity of the human cortex in order to confirm previously reported topological organisation patterns (network properties), such as small-worldness, modularity, hierarchy and core formation (see Figure 1), at both high- and low-resolution representations of cortical connectivity, and to then analyse the relationship of these patterns to the wiring constraints of the brain. To do so we devised two novel surrogate types, ‘spatial’ and ‘reduced’ surrogates that conserve the total length of connections from each region (spatial) or decrease it (reduced). For each network property, our analysis adopted the reasoning detailed in the *Introduction* (see also Table 1).

First, by comparing the cortical network and the spatially constrained surrogates to random surrogates, we found that cortical wiring constraints seem to contribute strongly to its relatively low potential for functional integration (as measured by global efficiency) and very high potential for functional segregation (as measured by clustering coefficient), and thus highly, although not fully (see below), account for the known small-world cortical organisation [57], [58]. In addition, comparison of the cortical connectivity network to the new surrogates also showed a relatively low level of global efficiency in the cortical network, closer to its reduced than to its spatial surrogates. Efficiency is a measure of functional integration potential in the network [15] and is generally most effectively increased by adding sparse long-range connections [18]. Because reduced surrogates to a great extent lack these long-range connections, our findings suggest that long-range cortico-cortical connections are in fact sub-optimally placed for maximising efficiency, and therefore, to the extent that brain structure determines function, they may not contribute to tight functional integration in the cortex as much as they could. In line with this, the cortical network was also found to be more similar to its reduced than to its spatial surrogates in its very high clustering coefficient. Functional segregation, facilitated by high structural clustering coefficient [15], is widely acknowledged to be a fundamental characteristic of the cortex [77]. Taken together, our findings indicate that the cortical network may possess an excess level of segregation and a relatively reduced level of functional integration potential over the extent that its wiring constraints alone can account for. Furthermore, spatial surrogates exhibited significantly weaker small-worldness compared to the cortical network, while reduced surrogates exhibited comparably high small-worldness to the cortical nework. These findings suggest that high cortical segregation combined with the concentrated spatial distribution of high degree regions (see Figure 6D) may suffice to ensure the strong small-world organisation of the cortical connectivity, even in the absence of long-range cortical connections.

Hierarchical organisation is believed to be a central architectural feature of various complex social networks and the World Wide Web [38] (Figure 1B). Hierarchical aspects of network architectures can fundamentally affect their evolution, development, adaptability and efficiency on multiple scales [61], [62]. The structural connectivity of the cortex is generally regarded to have a hierarchical organisation [59]. However, neither the degree and extent of hierarchical organisation, nor the constraints governing its expression, have yet been analysed in large-scale whole-brain networks as comprehensively as for instance the concepts of modularity or regional centrality [59]. This may be due to a lack of a consensus on the formal definition and assessment of this rather informal notion, in combination with currently available data being insufficiently detailed to evaluate and characterise the exact nature of this organisation pattern on a global scale [1], [78]. Here, we utilised the mean clustering coefficient as a function of degree, as a simple model for detecting hierarchical features in complex networks. The results indicated the presence of hierarchical organisation in the cortical network and in both spatially constrained surrogates, but not in random surrogates. One interpretation of this finding is that the predominantly local connectivity of the cortex and the central positioning of high degree regions give rise to the observed hierarchical structure. However, we cannot exclude an alternative explanation, namely that it is in fact the strong evolutionary pressure favouring the presumably functionally beneficial hierarchical organization, that led to the observed spatial embedding of cortical network nodes. Nevertheless, as both pressures, economical to conserve wiring cost and adaptive to achieve brain function, appear to benefit from a hierarchical organisation [18], [59], it seems most likely that their joint, mutual presence resulted in the observed hierarchical pattern in the structural connectivity of the cortex.

The brain's modular architecture is organised around spatially compact modules and their predominantly short, intra-module connections [77]. This feature of cortical connectivity is believed not only to keep global wiring costs low (economic pressure), but also to improve local communication efficiency within its structurally segregated and functionally specialised modular units (functional pressure) [18]. Our modularity analysis revealed that all spatially constrained networks indeed exhibit a strong and spatially compact modular architecture compared to random surrogates, indicating that basic wiring constraints of cortical regions naturally result in a tendency for cortical module formation. On the other hand, the high strength and granularity of the modular organisation of the cortex is more akin to its reduced surrogates, than to its relatively less modular spatial surrogates. This suggests that the long-range cortico-cortical projections may be more optimally placed towards a highly modular cortical architecture, than wiring constraints alone would suggest, supporting the widely acknowledged notion of high functional importance of cortical modules [21], [63], [64].

Furthermore, while the module partitions of the cortical network and its surrogates exhibit considerable differences, we found a set of cortical areas with modules that are highly preserved both in reduced and spatial surrogates. According to our analysis, the highly robust topological encapsulation of these predominantly lateral modules against the applied spatially constrained rewiring indicates that their existence can largely be explained by cortical wiring constraints. Additionally, however, the natural emergence of these module formations may enable them to provide a consistent base or ‘backbone’ to the cortex's modular structure both across individual variation and through development and ageing processes [24]. Such a modular ‘backbone’ structure could provide the structural basis for some relatively invariant, recurring components of the continuously reconfiguring functional networks of the brain [77].

While the exponential degree distribution [12] and hierarchical organisation already suggested a centralised organisation of cortical topology, we explicitly examined which, if any, parts of the cortex are located in its topological centre. Surrogate comparison revealed that the s-core of the high-resolution cortical network is stronger and larger than those of its spatial surrogates, and similar to those of its reduced surrogates. Furthermore, confirming previous results [12], the s-core of the cortical network was found to be spatially encapsulated at a medial-caudal location, composed by the precuneus, the cingulate cortex and the superior part of the occipital lobe. The cortical network, when observed on high-resolution (but not on low-resolution, see below), therefore appears to have a spatially compact, central s-core, the strength of which is significantly higher than its wiring constraints alone would suggest. One could interpret these findings to suggest that the cortical network s-core is *not* a by-product of wiring constraints but may instead be relevant for the brain's function; it might even serve the purpose of a putative central, global integrator substructure among the otherwise separate, functionally more specialised areas of the brain [79].

The other candidate central structure, the rich-club of the cortical network, also exhibits a significantly denser than random intra-connectedness, which is in agreement with previous studies detecting a well-expressed cortical rich-club structure [13], [17], [25]. However, in contrast to our results on the cortical s-core, we found rich-club structures of similar strength in the reduced and spatial surrogates. Thus, the rich-club formation of the cortical network appears to strongly correlate with its wiring constraints and the spatial distribution of the cortical hub regions (one of the ‘basic’ network property preserved in all surrogate ensembles). These findings extend earlier studies consistently discovering the brain's strong rich-club structure [13], [17], [25] by pointing to a plausible relationship between the remarkably dense inter-connectedness of high degree cortical regions and cortical wiring constraints. It is important to note, however, that similarly to the case of the hierarchy analysis, our method does not provide information with respect to the direction of causation between these network properties. Thus, it remains to be seen whether the economical pressure to conserve connection length is in fact the primary driving factor in the spatial arrangement of hub nodes, or the functional pressure for rich-club formation necessitates the specific spatial distribution of hub nodes in the cortex in the first place, and thus ultimately the formation of the cortical rich-club patterning.

Furthermore, compared to the s-core, the rich-club of the high-resolution cortical network was found to be internally relatively loosely coupled and formed by a spatially and topologically rather dispersed set of regions. These findings render the even spatially highly significant, well-confined and more tightly intra-connected cortical s-core a more appropriate candidate for a putative central cortical core [12], while the rich-club seems to be more suited for fulfilling the role of a ‘dynamic router’ [25], a set of distributed cortical hub regions predominantly connecting their local neighbourhoods with distant parts and the s-core of the cortex. Nevertheless, the large (50%) overlap between the s-core and rich-club regions suggests a great extent of functional cooperation between these highly intertwined, both topologically and spatially central cortical structures.

In line with these results, areas in the overlap between the s-core and rich-club structures of the cortex, the precuneus, the cingulate cortex and parts of the primary visual cortex (BA 17, 18), have also been repeatedly identified as global functional hubs of the human brain [80], [81], and found to functionally mediate between cortical areas that are structurally not directly connected [82]. Furthermore, some of the regions that belong to both the s-core and rich-club structures, most notably the precuneus, have also been highlighted as prominent areas of the default mode network of the brain [12], [83]. These findings suggest that the regions shared between the cortical network's s-core and rich-club, are not only topologically central, but also play a functionally pivotal role in coordinating, integrating or routing the activity of distant cortical regions in both resting and task-evoked states of the brain [25], [79], [83].

Figure 7 summarises the results on the investigated properties of the cortical network with respect to the three surrogate groups, at both network resolutions (998 regions at high-resolution and 66 regions at lower resolution), both for binary and weighted networks on each resolutions, as well as for the high-resolution weighted single hemisphere analysis (500 regions). First, comparing the metric values of the cortical network with those of its surrogates, we note that the cortical network tends to exhibit more similar values to its reduced than to its spatial surrogates for several network measures. One could argue that this may simply originate from the fact that the spatial surrogates are in general more randomised, and hence less similar to the cortical network, than reduced surrogates (see QS in Figure 7). However, while similarities in the expression of higher level network properties are certainly expected to be related to the extent of similarity on the lowest level of single connections, considering solely the overlap in the connection sets does not satisfactorily explain all observed tendencies. Indeed, as we showed in *Results/Topological similarity*, spatial surrogates are equally different from each other in their connection sets than from the cortical network, and yet their network properties are highly similar, but significantly different from that of the cortex. The overlap QS between connection sets alone is therefore not a good predictor of the obtained results, supporting our reasoning about the observed differences being attributable to the particular connectivity of the cortex – to its predominantly local connectivity *and* the specific arrangement of its long-range connections (see Table 1).

Secondly, Figure 7 assesses the consistency of our analyses across all investigated cortical network types (the five main rows of Figure 7). We start by noting that the results for several measures, most notably clustering coefficient, efficiency, small-worldness and modularity, are highly consistent across all investigated cortical network types. There is, however, some disagreement in the results of other complex network measures, specifically the k-core/s-core and rich-club metrics, across the various cortical networks. Generally, these disagreements indicate that the significance of the corresponding network properties (in terms of their relationship to the corresponding surrogate ensembles) may depend on the resolution the cortical network is observed at, or on the inclusion/exclusion of connection *strengths* (estimated number of fibres constituting the fibre bundles linking the regions), see detailed discussion in *Results*. Most notably, at the s-core/k-core metric, the strength of the cortical core only becomes visible in the high-resolution network, indicating a change in the organisation of the cortical connectivity at the different observable network resolutions and underlining the importance of multi-resolution approaches in connectome research. Specifically, on low resolution we found that the relatively weak cortical k-core is composed of 80% of the entire cortex, suggesting a more ‘homogeneous’ (non-centralised) connectivity between larger cortical regions on low network resolution. In contrast, on high-resolution the cortical network demonstrates a relatively small (10%), highly localised and significantly strong core structure, indicating a rather centralised organisation at the finer connectivity of the cortex. These findings are largely consistent with previous results on the s-core of the low-resolution [13] and high-resolution [12] cortical connectivity, and support the notion that, as we map the brain's network on increasingly higher resolutions, observed connectivity necessarily becomes sparser, leading in turn to the observation of fundamentally different organisation features at the various resolutions [78].

In this study, we focused on two distinguishable, supposedly competing factors that shape brain structure: economic pressure and functional pressure [18]. We note, however, that there are other important factors, such as evolutionary or developmental processes, that are likely to impose certain basic constraints on brain architecture [18]. Future extensions of this study may need to incorporate certain aspects of these further constraints, for example by generating surrogate networks via some neurobiologically informed developmental model [84]. It is also important to consider the accuracy of the cortical connectivity dataset used here. It is well known (and indeed increasingly articulated) that diffusion magnetic resonance imaging (dMRI) based tractography techniques suffer from certain biases and constraints, such as limitations in the ability to track fibre crossings and wide angular changes along the trajectory of the fibre tract [85], [86]. Therefore, in the current absence of comprehensive tract-tracing data in the human brain, it will be important that the hypotheses and computational findings of our study are tested against the increasingly complete and accurate maps dMRI techniques will be delivering in the future. Relatedly, it is likely that the spatially constrained surrogate analysis introduced in this study may give insights into the relative significance and potential origin of certain properties of the brain networks of other species, such as the cat [17] or the macaque [75], for which tract-tracing data is available.

Being a real complex network with a diverse and extraordinarily complex set of functions to carry out, it is not surprising that the cortex adopts and takes advantage of several functionally beneficial organisation patterns, even given the additional constraints imposed by wiring constraints [18]. *Small-world architecture* has been shown to naturally foster high dynamical complexity [9], [87], which is one of the hallmarks of brain activity [88] and has been associated with conscious states involving the efficient coordination of multiple sensorimotor modalities in generating flexible behaviour [89]. *Modularity* is widely acknowledged to promote network robustness and evolvability by minimising dependencies and isolating the effect of local mutations and disturbances [2]. It also has been shown to increase dynamical metastability [63] thus hindering the pathological cases of prolonged synchronisation and seizures [90] and again supporting functional flexibility [91]. *Hierarchically modular organisation* has been found to facilitate limited sustained network activity [92], it hence may serve a crucial role in maintaining the critical functional range in which the human brain operates [93]. Furthermore, the strong *central core* as well as the distributed and yet densely inter- and intra-connected *rich-club* structure may play a central role in facilitating efficient global functional integration and information flow in the cortex [13], [25], [74] hence providing the structural basis of various cognitive integration processes, from sensorimotor integration through attention to higher cognition and consciousness [77], [94]. Combining all these findings into a single description of the structural connectivity of the human cortex, our results outline a hybrid, reasonably centralised and hierarchical, but nevertheless strongly modular anatomical architecture, with a remarkably strong central network core.

Consistent discovery of characteristic network properties of the human connectome in this and previous studies emphasises a fundamental question: What factors contribute to the small-world, modular, hierarchical and centralised features of the cortical connectivity? Our results, extending those of earlier studies [51], [95], [96], support the notion that the emergence of these network properties is shaped by a complex interaction involving economic pressures (towards minimising wiring and running cost of the brain) and functional pressures (towards stable, reliable and adaptive operation of the brain) [18]. In this study we characterised how much each specific network property depended on the first of these factors, economic pressures, and we found that the level of dependency differed for different properties. Our results suggest that the more independent properties, such as the small-world, modular and core structure of the cortex, may be more related to the function of the brain than the more dependent ones, such as hierarchical organisation and rich-club patterning, which may be primarily driven by economic pressures. These results motivate further computational and experimental research to uncover the specific ways in which economic and functional pressures complement, reinforce or counteract each other in shaping the large-scale architecture of the human brain.

**Box 1**

- **Cortical connectome/cortical connectivity** A network (or graph) representation of the large-scale white-matter pathways of the cortex, with cortical grey matter regions as network nodes, and their connecting white-matter fibre bundles as edges.
- **Spatial network** A network that is physically embedded into (some dimensions of) the three dimensional space of the world. For example, electric power grids and flight networks are two dimensional spatial networks (geographical networks), brain networks are three dimensional spatial networks, but gene regulatory networks, word networks and the World Wide Web are non-spatial (abstract or ‘symbolic’) networks.
- **Binary/weighted network** A network is binary if its links only denote the presence or absence of connections, while in a weighted network links also contain information about the strength of connections.
- **Functional pressure** An evolutionary and/or adaptive pressure on the brain to produce behavioural patterns beneficial for survival.
- **Economic pressure** An evolutionary and/or developmental pressure on the brain to minimise its wiring and running costs.
- **Wiring constraints** Constraints on the connectivity of the brain arising from its embedding in the limited space of the skull as well as from the high developmental and metabolic costs of long-range connections (economic pressure).
- **Surrogate networks** A ‘null-hypothesis’ network ensemble that is used to test by comparison the significance of certain network properties of the original network with respect to a set of its ‘basic’ properties, such as size and connection density.
- **Random surrogates** An ensemble of surrogate networks obtained by ‘unconstrained’ randomization. Random surrogates only preserve the size, connection density and the degree distribution of the cortical network.
- **Spatial surrogates** An ensemble of surrogate networks obtained by ‘wiring-length-conserving’ randomization. Spatial surrogates preserve the size, connection density and the degree distribution of the cortical network, and also conserve its wiring length distribution.
- **Reduced surrogates** An ensemble of surrogate networks obtained by ‘wiring-length-decreasing’ randomization. Reduced surrogates preserve the size, connection density and the degree distribution of the cortical network, while further reducing the wiring lengths as close as possible to the minimum.
- **Network property** A specific characteristic of the organisation of the network, such as small-worldness or modularity.
- **Complex network measure** A formal (mathematical) metric devised to measure the extent of expression of a certain network property.
- **Network segregation** Potential for specialised processing to occur within distributed and usually densely intra-connected parts of a network.
- **Network integration** Potential for efficiently exchange and combine specialised information from distributed parts of a network.

## References

[1] Sporns O, Tononi G, Kötter R (2005) The human connectome: A structural description of the human brain. PLoS Comput Biol 1–4 doi:10.1371/journal.pcbi.0010042 10.1371/journal.pcbi.0010042 PMC1239902 16201007

[2] Sporns O (2010) Networks of the Brain. The MIT Press. 424p.

[3] Bressler SL, Menon V (2010) Large-scale brain networks in cognition: emerging methods and principles. Trends Cogn Sci 14 6: 277–90. 20493761 10.1016/j.tics.2010.04.004

[4] Conturo TE, Lori NF, Cull TS, Akbudak E, Snyder AZ, et al (1999) Tracking neuronal fiber pathways in the living human brain. Proc Natl Acad Sci U S A 96 18: 10422–10427. 10468624 10.1073/pnas.96.18.10422 PMC17904

[5] Mori S, Crain BJ, Chacko VP, van Zijl PC (1999) Three-dimensional tracking of axonal projections in the brain by magnetic resonance imaging. Ann Neurol 45 2: 265–269. 9989633 10.1002/1531-8249(199902)45:2<265::aid-ana21>3.0.co;2-3

[6] Johansen-Berg H, Rushworth MF (2009) Using diffusion imaging to study human connectional anatomy. Annu Rev Neurosci 32: 75–94. 19400718 10.1146/annurev.neuro.051508.135735

[7] Pan H, Epstein J, Silbersweig DA, Stern E (2011) New and emerging imaging techniques for mapping brain circuitry. Brain Res Rev 67 1–2: 226–251. 21354205 10.1016/j.brainresrev.2011.02.004

[8] Cammoun L, Gigandet X, Meskaldji D, Thiran JP, Sporns O, et al (2012) Mapping the human connectome at multiple scales with diffusion spectrum MRI. J Neurosci Methods 203 2: 386–397. 22001222 10.1016/j.jneumeth.2011.09.031

[9] Sporns O, Tononi G, Edelman GM (2000) Theoretical neuroanatomy: relating anatomical and functional connectivity in graphs and cortical connection matrices. Cereb Cortex 10 2: 127–141. 10667981 10.1093/cercor/10.2.127

[10] Bassett DS, Bullmore E, Verchinski BA, Mattay VS, Weinberger DR, et al (2008) Hierarchical organisation of human cortical networks in health and schizophrenia. J Neurosci 28 37: 9239–9248. 18784304 10.1523/JNEUROSCI.1929-08.2008 PMC2878961

[11] Hilgetag CC, Burns GA, O'Neill MA, Scannell JW, Young MP (2000) Anatomical connectivity defines the organisation of clusters of cortical areas in the macaque monkey and the cat. Philos Trans R Soc Lond B Biol Sci 355 1393: 91–110. 10703046 10.1098/rstb.2000.0551 PMC1692723

[12] Hagmann P, Cammoun L, Gigandet X, Meuli R, Honey CJ, et al (2008) Mapping the structural core of human cerebral cortex. PLoS Biol 6 7, doi:10.1371/journal.pbio.0060159 10.1371/journal.pbio.0060159 PMC2443193 18597554

[13] Van den Heuvel MP, Sporns O (2011) Rich-club organisation of the human connectome. J Neurosci 31 44: 15775–15786. 22049421 10.1523/JNEUROSCI.3539-11.2011 PMC6623027

[14] Milo R, Shen-Orr S, Itzkovitz S, Kashtan N, Chklovskii D, et al (2002) Network motifs: simple building blocks of complex networks. Science 298 5594: 824–827. 12399590 10.1126/science.298.5594.824

[15] Rubinov M, Sporns O (2010) Complex network measures of brain connectivity: Uses and interpretations. Neuroimage 52 3: 1059–69. 19819337 10.1016/j.neuroimage.2009.10.003

[16] Boccaletti S, Latora V, Moreno Y, Chavez M, Hwang D (2006) Complex networks: Structure and dynamics. Physics Reports 424 4–5: 175–308.

[17] Zamora-López G, Zhou C, Kurths J (2010) Cortical hubs form a module for multisensory integration on top of the hierarchy of cortical networks. Front Neuroinformatics 4: 1. 10.3389/neuro.11.001.2010 PMC2859882 20428515

[18] Bullmore E, Sporns O (2012) The economy of brain network organization. Nat Rev Neurosci 13 5: 336–49. 22498897 10.1038/nrn3214

[19] Braitenberg V and Schuz A (1998) Cortex: Statistics and Geometry of Neuronal Connectivity. Springer-Verlag 2nd edition. 249p.

[20] Sporns O, Kötter R (2004) Motifs in Brain Networks. PLoS Biol 2 11, doi:10.1371/journal.pbio.0020369 10.1371/journal.pbio.0020369 PMC524253 15510229

[21] Sporns O (2006) Small-world connectivity, motif composition, and complexity of fractal neuronal connections. Biosystems 85 1: 55–64. 16757100 10.1016/j.biosystems.2006.02.008

[22] Sporns O, Honey CJ, Kötter R (2007) Identification and classification of hubs in brain networks. PLoS One 2 10, doi:10.1371/journal.pone.0001049 10.1371/journal.pone.0001049 PMC2013941 17940613

[23] Watts DJ, Strogatz SH (1998) Collective dynamics of ‘small-world’ networks. Nature 393 6684: 440–442. 9623998 10.1038/30918

[24] Echtermeyer C, Han CE, Rotarska-Jagiela A, Mohr H, Uhlhaas PJ, Kaiser M (2011) Integrating temporal and spatial scales: human structural network motifs across age and region of interest size. Front Neuroinform 5: 10 doi:10.3389/fninf.2011.00010 21811454 10.3389/fninf.2011.00010 PMC3143730

[25] van den Heuvel MP, Kahn RS, Goñi J, Sporns O (2012) High-cost, high-capacity backbone for global brain communication. Proc Natl Acad Sci U S A 109 28: 11372–7. 22711833 10.1073/pnas.1203593109 PMC3396547

[26] Cabral J, Hugues E, Sporns O, Deco G (2011) Role of local network oscillations in resting-state functional connectivity. Neuroimage 57 1: 130–139. 21511044 10.1016/j.neuroimage.2011.04.010

[27] Honey CJ, Sporns O, Cammoun L, Gigandet X, Thiran JP, et al (2009) Predicting human resting-state functional connectivity from structural connectivity. Proc Natl Acad Sci U S A 106 6: 2035–2040. 19188601 10.1073/pnas.0811168106 PMC2634800

[28] Shanahan M, Wildie M (2012) Knotty-Centrality: Finding the Connective Core of a Complex Network. PLoS ONE 7 5, doi:10.1371/journal.pone.0036579 10.1371/journal.pone.0036579 PMC3348887 22590571

[29] Fornito A, Zalesky A, Bullmore ET (2010) Network scaling effects in graph analytic studies of human resting-state fmri data. Front Syst Neurosci 4: 22–22. 20592949 10.3389/fnsys.2010.00022 PMC2893703

[30] Zalesky A, Fornito A, Harding IH, Cocchi L, Yücel M, et al (2010) Whole-brain anatomical networks: does the choice of nodes matter? Neuroimage 50 3: 970–983. 20035887 10.1016/j.neuroimage.2009.12.027

[31] Maslov S, Sneppen K (2002) Specificity and stability in topology of protein networks. Science 296 5569: 910–913. 11988575 10.1126/science.1065103

[32] Sørensen T (1948) A method of establishing groups of equal amplitude in plant sociology based on similarity of species content. Kongelige Danske Videnskabernes Selskab Biol 4: 1–34.

[33] Latora V, Marchiori M (2001) Efficient behavior of small-world networks. Phys Rev Lett 87 19: 198701–198701. 11690461 10.1103/PhysRevLett.87.198701

[34] Achard S, Bullmore E (2007) Efficiency and cost of economical brain functional networks. PLoS Comput Biol 3 2, doi:10.1371/journal.pcbi.0030017 10.1371/journal.pcbi.0030017 PMC1794324 17274684

[35] Onnela JP, Saramäki J, Kertész J, Kaski K (2005) Intensity and coherence of motifs in weighted complex networks. Phys Rev E Stat Nonlin Soft Matter Phys 71 6 Pt 2, doi:10.1103/physreve.71.065103 10.1103/PhysRevE.71.065103 16089800

[36] Sporns O (2011) The non-random brain: efficiency, economy, and complex dynamics. Front Comput Neurosci 5: 5–5. 21369354 10.3389/fncom.2011.00005 PMC3037776

[37] Humphries MD, Gurney K (2008) Network ‘small-world-ness’: a quantitative method for determining canonical network equivalence. PLoS One 3 4, doi:10.1371/journal.pone.0002051 10.1371/journal.pone.0002051 PMC2323569 18446219

[38] Ravasz E, Barabási AL (2003) Hierarchical organisation in complex networks. Phys Rev E Stat Nonlin Soft Matter Phys 67 2 Pt 2: 026112–026112. 12636753 10.1103/PhysRevE.67.026112

[39] Newman ME (2004) Fast algorithm for detecting community structure in networks. Phys Rev E Stat Nonlin Soft Matter Phys 69 6 Pt 2: 066133–066133. 15244693 10.1103/PhysRevE.69.066133

[40] Danon L, Díaz-Guilera A, Duch J, Arenas A (2005) Comparing community structure identification. Journal of Statistical Mechanics: Theory and Experiment 2005 09: P09008.

[41] Newman ME (2006) Modularity and community structure in networks. Proc Natl Acad Sci U S A 103 23: 8577–8582. 16723398 10.1073/pnas.0601602103 PMC1482622

[42] Steen M, Hayasaka S, Joyce K, Laurienti P (2011) Assessing the consistency of community structure in complex networks. Phys Rev E Stat Nonlin Soft Matter Phys Jul; 84 1 Pt 2: 016111. 10.1103/PhysRevE.84.016111 PMC3292265 21867261

[43] Meila M (2007) Comparing clusterings – an information based distance. J Multivar Anal 98 5: 873–895.

[44] Karrer B, Levina E, Newman ME (2008) Robustness of community structure in networks. Phys Rev E Stat Nonlin Soft Matter Phys 77 4 Pt 2, doi:10.1103/physreve.77.046119 10.1103/PhysRevE.77.046119 18517702

[45] Traud AL, Kelsic ED, Mucha PJ, Porter MA (2011) Comparing Community Structure to Characteristics in Online Collegiate Social Networks. SIAM Review 53 3: 526–543.

[46] Seidman SB (1983) Network Structure and Minimum Degree. Social Networks 5: 269–287.

[47] Zhou S, Mondragón RJ (2004) The rich-club phenomenon in the internet topology. IEEE Comm Lett 8 3: 180–182.

[48] Colizza V, Flammini A, Serrano MA, Vespignani A (2006) Detecting rich-club ordering in complex networks. Nat Phys 2 2: 110–115.

[49] Opsahl T, Colizza V, Panzarasa P, Ramasco JJ (2008) Prominence and control: the weighted rich-club effect. Phys Rev Lett 101 16: 168702–16870 2. 18999722 10.1103/PhysRevLett.101.168702

[50] Newman MEJ (2002) Assortative mixing in networks. Phys Rev Lett 89 20: 208701. 12443515 10.1103/PhysRevLett.89.208701

[51] Kaiser M, Hilgetag CC (2006) Nonoptimal component placement, but short processing paths, due to long-distance projections in neural systems. PLoS Comput Biol 2 7, doi:10.1371/journal.pcbi.0020095 10.1371/journal.pcbi.0020095 PMC1513269 16848638

[52] Ahn YY, Jeong H, Kim BJ (2006) Wiring cost in the organization of a biological neuronal network. Physica A 367: 531–537.

[53] Bassett DS, Greenfield DL, Meyer-Lindenberg A, Weinberger DR, Moore SW, Bullmore ET (2010) Efficient physical embedding of topologically complex information processing networks in brains and computer circuits. PLoS Comput Biol 6 4, doi:10.1371/journal.pcbi.1000748 10.1371/journal.pcbi.1000748 PMC2858671 20421990

[54] Chen Y, Wang S, Hilgetag CC, Zhou C (2013) Trade-off between multiple constraints enables simultaneous formation of modules and hubs in neural systems. PLoS Comput Biol 9 3, doi:10.1371/journal.pcbi.1002937 10.1371/journal.pcbi.1002937 PMC3591279 23505352

[55] Tononi G, Sporns O, Edelman GM (1994) A measure for brain complexity: relating functional segregation and integration in the nervous system. Proc Natl Acad Sci USA 91 11: 5033–5037. 8197179 10.1073/pnas.91.11.5033 PMC43925

[56] Humphries MD, Gurney K, Prescott TJ (2006) The brainstem reticular formation is a small-world, not scale-free, network. Proceedings of the Royal Society B: Biological Sciences 273 1585: 503–511. 16615219 10.1098/rspb.2005.3354 PMC1560205

[57] Sporns O, Honey CJ (2006) Small worlds inside big brains. Proc Natl Acad Sci USA 103 51: 19219–19220. 17159140 10.1073/pnas.0609523103 PMC1748207

[58] Bassett DS, Bullmore E (2006) Small-world brain networks. Neuroscientist 12 6: 512–523. 17079517 10.1177/1073858406293182

[59] Kaiser M, Hilgetag CC, Kotter R (2010) Hierarchy and dynamics of neural networks. Frontiers in Neuroinformatics 4 0: 112 doi:10.3389/fninf.2010.00112 20844605 10.3389/fninf.2010.00112 PMC2938979

[60] Newman MEJ, Girvan M (2004) Finding and evaluating community structure in networks. Phys Rev E 69 2: 026113. 10.1103/PhysRevE.69.026113 14995526

[61] Simon HA (1962) The architecture of complexity. In Proceedings of the American Philosophical Society volume 106 pages 467–482.

[62] Koch C, Laurent G (1999) Complexity and the nervous system. Science 284 5411: 96–98. 10102826 10.1126/science.284.5411.96

[63] Shanahan M (2010) Metastable chimera states in community-structured oscillator networks. Chaos 20 1: 013108–013108. 20370263 10.1063/1.3305451

[64] Kaiser M, Goerner M, Hilgetag CC (2008) Criticality of spreading dynamics in hierarchical cluster networks without inhibition. New Journal of Physics 9 5: 110–110.

[65] Chen ZJ, He Y, Rosa-Neto P, Germann J, Evans AC (2008) Revealing modular architecture of human brain structural networks by using cortical thickness from mri. Cereb Cortex 18 10: 2374–2381. 18267952 10.1093/cercor/bhn003 PMC2733312

[66] Valencia M, Pastor MA, Fernández-Seara MA, Artieda J, Martinerie J, et al (2009) Complex modular structure of large-scale brain networks. Chaos 19 2: 023119–023119. 19566254 10.1063/1.3129783

[67] Meunier D, Lambiotte R, Fornito A, Ersche KD, Bullmore ET (2009b) Hierarchical modularity in human brain functional networks. Front Neuroinform 3: 37–37. 19949480 10.3389/neuro.11.037.2009 PMC2784301

[68] He Y, Wang J, Wang L, Chen ZJ, Yan C, et al (2009) Uncovering intrinsic modular organisation of spontaneous brain activity in humans. PLoS One 4 4, doi:10.1371/journal.pone.0005226 10.1371/journal.pone.0005226 PMC2668183 19381298

[69] Meunier D, Achard S, Morcom A, Bullmore E (2009a) Age-related changes in modular organisation of human brain functional networks. Neuroimage 44 3: 715–723. 19027073 10.1016/j.neuroimage.2008.09.062

[70] Borgatti SP, Everett MG (2000) Models of Core/Periphery Structures. Social Networks 21 4: 375–395.

[71] Rossa FD, Dercole F, Piccardi C (2013) Profiling core-periphery network structure by random walkers. Sci Rep 3: 1467. 23507984 10.1038/srep01467 PMC3601366

[72] Rombach MP, Porter MA, Fowler JH, Mucha PJ (2013) Core-Periphery Structure in Networks, submitted to SIAM Journal on Applied Mathematics, arXiv:1202.2684.

[73] Zamora-López G, Zhou C, Kurths J (2009) Graph analysis of cortical networks reveals complex anatomical communication substrate. Chaos 19 1: 015117–015117. 19335021 10.1063/1.3089559

[74] Gómez-Gardeñes J, Zamora-López G, Moreno Y, Arenas A (2010) From modular to centralized organization of synchronization in functional areas of the cat cerebral cortex. PLoS One 5 8, doi:10.1371/journal.pone.0012313 10.1371/journal.pone.0012313 PMC2928734 20865046

[75] Modha DS, Singh R (2010) Network architecture of the long-distance pathways in the macaque brain. Proc Natl Acad Sci U S A 107 30: 13485–13490. 20628011 10.1073/pnas.1008054107 PMC2922151

[76] Kaiser M, Varier S (2011) Evolution and development of brain networks: from Caenorhabditis elegans to Homo sapiens. Network 22 1–4: 143–7. 22149674 10.3109/0954898X.2011.638968

[77] Sporns O (2013) Network attributes for segregation and integration in the human brain. Curr Opin Neurobiol 23 2: 162–71. 23294553 10.1016/j.conb.2012.11.015

[78] Sporns O (2013) The human connectome: Origins and challenges. NeuroImage 80: 53–61. 23528922 10.1016/j.neuroimage.2013.03.023

[79] Baars BJ (2002) The conscious access hypothesis: origins and recent evidence. Trends Cogn Sci 6 1: 47–52. 11849615 10.1016/s1364-6613(00)01819-2

[80] Tomasi D, Volkow ND (2011) Association between functional connectivity hubs and brain networks. Cereb Cortex 21 9: 2003–13. 21282318 10.1093/cercor/bhq268 PMC3165965

[81] Tomasi D, Volkow ND (2011) Functional connectivity hubs in the human brain. Neuroimage 57 3: 908–17. 21609769 10.1016/j.neuroimage.2011.05.024 PMC3129362

[82] Greicius MD, Supekar K, Menon V, Dougherty RF (2009) Resting-state functional connectivity reflects structural connectivity in the default mode network. Cereb Cortex 19 1: 72–8. 18403396 10.1093/cercor/bhn059 PMC2605172

[83] Raichle ME, Snyder AZ (2007) A default mode of brain function: a brief history of an evolving idea. Neuroimage 37 4: 1083–90. 17719799 10.1016/j.neuroimage.2007.02.041

[84] Jbabdi S, Johansen-Berg H (2011) Tractography: where do we go from here? Brain Connect 1 3: 169–183. 22433046 10.1089/brain.2011.0033 PMC3677805

[85] Sporns O, Chialvo DR, Kaiser M, Hilgetag CC (2004) Organization, development and function of complex brain networks. Trends Cogn Sci 8 9: 418–25. 15350243 10.1016/j.tics.2004.07.008

[86] Van Essen DC (1997) A tension-based theory of morphogenesis and compact wiring in the central nervous system. Nature 385 6614: 313–8. 9002514 10.1038/385313a0

[87] Shanahan M (2008) Dynamical complexity in small-world networks of spiking neurons. Phys Rev E Stat Nonlin Soft Matter Phys 78 4 Pt 1, doi:10.1103/PhysRevE.78.041924 10.1103/PhysRevE.78.041924 18999472

[88] Stam CJ (2005) Nonlinear dynamical analysis of EEG and MEG: review of an emerging field. Clin Neurophysiol 116 10: 2266–301. 16115797 10.1016/j.clinph.2005.06.011

[89] Seth AK, Barrett AB, Barnett L (2011) Causal density and integrated information as measures of conscious level. Phil Trans R Soc A 369: 3748–3767. 21893526 10.1098/rsta.2011.0079

[90] Arthuis M, Valton L, Régis J, Chauvel P, Wendling F, et al (2009) Impaired consciousness during temporal lobe seizures is related to increased long-distance cortical-subcortical synchronization. Brain 132 Pt 8: 2091–2101. 19416952 10.1093/brain/awp086

[91] Bhowmik D, Shanahan M (2013) Metastability and inter-band frequency modulation in networks of oscillating spiking neuron populations. PLoS One 8 4, doi:10.1371/journal.pone.0062234 10.1371/journal.pone.0062234 PMC3628585 23614040

[92] Kaiser M, Hilgetag CC (2010) Optimal hierarchical modular topologies for producing limited sustained activation of neural networks. Front Neuroinform 4: 8. 20514144 10.3389/fninf.2010.00008 PMC2876872

[93] Kitzbichler MG, Smith ML, Christensen SR, Bullmore E (2009) Broadband Criticality of Human Brain Network Synchronization. PLoS Comput Biol 5 3, doi:10.1371/journal.pcbi.1000314 10.1371/journal.pcbi.1000314 PMC2647739 19300473

[94] Shanahan M (2012) The brain's connective core and its role in animal cognition. Phil Trans Roy Soc B 367: 2704–2714. 22927569 10.1098/rstb.2012.0128 PMC3427545

[95] Chklovskii DB (2004) Synaptic connectivity and neuronal morphology: two sides of the same coin. Neuron 43: 609–617. 15339643 10.1016/j.neuron.2004.08.012

[96] Rubinov M, Sporns O, van Leeuwen C, Breakspear M (2009) Symbiotic relationship between brain structure and dynamics. BMC Neurosci 10: 55. 19486538 10.1186/1471-2202-10-55 PMC2700812

[97] Holten D (2006) Hierarchical edge bundles: visualization of adjacency relations in hierarchical data. IEEE Transactions on Visualization and Computer Graphics 12 5: 741–748. 17080795 10.1109/TVCG.2006.147

